# The First Report of a Bang-Sensitive Strain in *Drosophila funebris*: Molecular-Genetic and Lipidomic Characterization

**DOI:** 10.3390/ijms27198845

**Published:** 2026-10-03

**Authors:** Karina A. Kosherova, Dmitrii A. Abashkin, Natalya B. Zakharzhevskaya, Victoriia D. Kazakova, Elizaveta A. Vorobyeva, Alexander V. Symonenko, Dmitry V. Mukha

**Affiliations:** 1 Vavilov Institute of General Genetics Russian Academy of Sciences, Gubkin St. 3, 119991 Moscow, Russia; 2 Mental Health Research Center, Clinical Genetics Laboratory, Kashirskoe Sh. 34, 115522 Moscow, Russia; 3 Department of Postgenomics, Lopukhin Federal Research and Clinical Center of Physical-Chemical Medicine, Federal Medical Biological Agency, 119435 Moscow, Russia; 4 National Research Centre “Kurchatov Institute”, 2 Kurchatov Sq., 123182 Moscow, Russia

**Keywords:** *Drosophila funebris*, bang sensitivity, transcriptomics, *Acox57D-d*, peroxisomal β-oxidation, lipidomics, seizure-and-paralysis susceptibility, cardiolipin, triacylglycerol accumulation, insect epilepsy model, thanatosis

## Abstract

Bang sensitivity (BS) is a well-characterized seizure-and-paralysis phenotype in *Drosophila melanogaster*, but its genetic basis remains unexamined in the phylogenetically distant sister species *Drosophila funebris*. Here, we identify a naturally occurring BS strain in *D. funebris* from a wild population near Lake Valday, characterized by acute mechanical shock-induced seizures and paralysis, and examine its molecular-genetic and lipidomic basis. Comparative transcriptomics identified 241 differentially expressed genes (FDR-adjusted *p* < 0.05), with “fatty acid metabolic process” as the most enriched GO term. Notably, within this pathway, the peroxisomal acyl-CoA oxidase gene *Acox57D-d* showed the most significant downregulation (log_2_*FC* = −4.21, *p*_adj_ = 4.1 × 10^−110^), validated by head-specific RT-qPCR. Untargeted lipidomics of BS heads (LC-Q-TOF-MS) revealed 145 altered lipid species, dominated by oxygenated triacylglycerol accumulation, cardiolipin/phosphatidylethanolamine depletion, and a divergent shift—depletion of DG 29:0 and accumulation of DG 47:4, reflecting impaired peroxisomal β-oxidation and potential mitochondrial and Na^+^/K^+^-ATPase dysfunction. These findings nominate *Acox57D-d* as a leading candidate gene associated with the phenotype and molecular signature of the studied BS line and establish this natural BS strain as a potentially useful model for investigating the relationship between peroxisomal lipid metabolism, neuronal excitability, and insect thanatosis.

## 1. Introduction

The bang sensitivity (BS) phenotype in *Drosophila melanogaster* is defined as the propensity of flies to enter a convulsive–paralytic state following mechanical stimulation. The first systematic classification of BS mutants was carried out by Ganetzky and Wu in 1982, who described a characteristic stereotyped behavioral sequence associated with mutations in specific genes and introduced the term “bang-sensitive” [1]. The initially characterized BS loci include *bss* (*bangsenseless*), *eas* (*easily shocked*), *tko* (*technical knockout*), *bas* (*bang-sensitive*), and *sda* (*slamdance*). Despite a uniform behavioral phenotype, no common biochemical defect has been identified across all mutants. Following mechanical stimulation, most BS mutants display a reproducible sequence of behavioral changes: an initial paroxysm (seizure), paralysis lasting approximately 1–2 min, and subsequent recovery. Subsequent work demonstrated that seizures and paralysis represent independent physiological events, and that motionless paralysis can occur in the absence of an overt seizure phase, often serving as the predominant manifestation of the phenotype [2].

According to the FlyBase database, over a hundred genes are associated with an epileptic phenotype in *D. melanogaster* [3]. While mutations in all of these genes do not necessarily produce the classical BS phenotype in the strict sense, loss of their function can significantly enhance or suppress it [4,5], consistent with the polygenic heterogeneity model [6]. The contribution of polygenic interactions to BS phenotype expressivity in *D. melanogaster* was first demonstrated experimentally at the population level in 2000 [7].

On the basis of genetic criteria, BS mutants can be divided into two categories: (a) primary (“classical”) BS loci, mutations which independently reproduce the behavioral sequence of seizures and/or paralysis, and (b) modifier loci, which alter phenotype expressivity and penetrance when combined with a primary BS allele. The primary BS genes of *D. melanogaster* identified to date, along with the functional classification of their respective mutations, are summarized in Supplementary Table S1 [1,8,9,10,11,12,13,14,15,16,17,18,19,20].

Notably, of 145 human genes associated with epilepsy, approximately 81% have orthologs in *D. melanogaster*, establishing *Drosophila* as a powerful translational model for investigating the molecular-genetic mechanisms of human seizure disorders [3,4,21,22,23,24] and for high-throughput screening of antiepileptic therapeutic agents [25,26,27].

Beyond direct epilepsy modeling, BS mutants may represent a valuable system for investigating the molecular mechanisms of thanatosis—reflex immobilization elicited by mechanical stimulation or predator threat, which is a conserved adaptive trait widely distributed among insects [28]. This notion aligns with the evolutionary “upside of epilepsy” hypothesis, which proposes that certain seizure-susceptibility alleles are maintained in natural populations because, at subthreshold doses, they confer an adaptive behavioral response to environmental stress [29]. In this context, a natural BS strain arising without artificial mutagenesis is of paramount interest for testing evolutionary and metabolic hypotheses.

In the present study, we describe for the first time a bang-sensitive strain of *Drosophila funebris* derived from the offspring of a single wild-caught female collected from a natural population on the shore of Lake Valday. *D. funebris* (Fabricius, 1787) belongs to the *funebris* group of the subgenus *Drosophila* and is among the most widespread synanthropic species of the genus. Unlike *D. melanogaster* (subgenus *Sophophora*), *D. funebris* exhibits distinct morphological, ecological, and physiological characteristics. Morphologically, *D. funebris* is distinguished by a considerably larger body size. More importantly, from an evolutionary and metabolic standpoint, *D. funebris* occupies a highly divergent ecological niche. While *D. melanogaster* is a tropical-origin species strictly adapted to fermenting sugary fruits, *D. funebris* is a cold-tolerant Holarctic species capable of exploiting a broad spectrum of decaying organic matter, including macrofungi, sap flows, and decomposing vegetables [30].

Historically, it was in this species that N. V. Timoféeff-Ressovsky developed the foundational concepts of penetrance and expressivity [31]. Despite its ecological significance and its role as the nominal type species of the genus *Drosophila*, *D. funebris* was subsequently largely displaced from model research. Consequently, *D. funebris* remains genetically understudied; while a chromosome-level *de novo* genome assembly has been recently sequenced and made publicly available [32], a comprehensive structural and functional gene annotation was completely lacking in public repositories. Gene mapping and transcript identification in this study were therefore performed using the *Liftoff* tool [33], transferring functional annotations from the high-quality reference genome of *D. melanogaster*.

The considerable phylogenetic distance separating *D. funebris* from *D. melanogaster* renders this species particularly valuable for comparative analysis: molecular mechanisms conserved across such an evolutionary span are strong candidates for functional significance extending well beyond any single model organism. Moreover, the use of a natural BS strain affords a unique opportunity to characterize the metabolic basis of the BS phenotype as it arises spontaneously in an outbred population.

The aims of the present study were: (1) to characterize the BS phenotype in a natural population of *D. funebris* and identify associated molecular-genetic changes by comparative transcriptomic analysis, with key findings verified by RT-qPCR; and (2) to perform comparative lipidomic profiling of BS and control *D. funebris* strains, motivated by transcriptomic evidence of altered lipid metabolism. By analyzing shifts in critical structural lipid species such as triacylglycerols, mitochondrial cardiolipin and phosphatidylethanolamine, as well as diacylglycerols, we aimed to evaluate the systemic effects of peroxisomal deficiency.

## 2. Results

### 2.1. Collection and Genetic Identification of Drosophilidae from Natural Populations

For over a decade, annual collections of Drosophilidae have been conducted in the geographic region of Lake Valday. To date, 13 species of the genus *Drosophila* have been identified, representing all three recognized subgenera: *Sophophora* (*D. melanogaster*, *D. obscura*, *D. subobscura*, *D. bifasciata*), *Drosophila* (*D. immigrans*, *D. funebris*, *D. testacea*, *D. transversa*, *D. phalerata*, *D. histrio*, *D. hydei*, *D. mercatorum*), and *Dorsilopha* (*D. busckii*). In addition, two species of the genus *Chymomyza* (*C. costata* and *C. amoena*) and *Scaptomyza* (*Parascaptomyza*) *pallida* were recorded.

Species identification was verified by sequencing fragments of the mitochondrial cytochrome c oxidase subunit I (*COI*) gene according to the Barcode of Life Data Systems (BOLD) platform protocols [34,35]. Only lines established from individual wild-caught females (isofemale lines, IFL) and successfully adapted to laboratory culture were included in subsequent analyses. All identified species are currently maintained as live cultures at the Laboratory of the Genetic Basis of Biodiversity, Vavilov Institute of General Genetics, Russian Academy of Sciences, with each species represented by at least three independent IFLs. Molecular barcoding data have been deposited on the BOLD platform under the dedicated DRORU project framework (https://portal.boldsystems.org/result?query=DRORU) (accessed on 2 September 2026). This ongoing monitoring effort serves a dual objective: the systematic collection and genetic identification of Drosophilidae across defined geographic localities, and the long-term maintenance of live IFL repositories to enable the detailed study of intra- and interpopulation metabolic and genetic variability.

### 2.2. The Bang-Sensitive Phenotype of Drosophila funebris

When comparing isofemale lines (IFL) of *Drosophila funebris* established from wild-caught females collected at the same geographic location (58.0248° N, 33.2351° E; approximately 100 m from the shore of Lake Valday) in 2018, one specific line exhibited pronounced hypersensitivity to mechanical stimulation. The bang-sensitive (BS) phenotype was first noticed in this line in its second or third generation after establishment and has remained stable over all subsequent generations of laboratory maintenance to date. Throughout the present study, this single BS line and a single control line of the same geographic origin were used consistently. Gentle shaking of the culture vial elicited a brief seizure-like paroxysm followed by prolonged paralysis (Video S1). The initial seizure-like phase was relatively short (approximately 15–20 s) and manifested as uncoordinated hyperkinetic movements and subsequent convulsive leg contractions. The ensuing immobile (paralytic) phase lasted approximately 2–2.5 min before spontaneous recovery, closely resembling the classical bang-sensitive (BS) phenotype described in *D. melanogaster*. In contrast, control *D. funebris* flies of identical geographic origin showed no behavioral response to equivalent mechanical stimulation. Direct comparison of mitochondrial *COI* gene fragment sequences between *D. funebris* lines with BS and normal phenotypes (GenBank accessions PZ780592 and PZ780593, respectively) revealed only a single nucleotide substitution across a region exceeding 600 nucleotides, confirming that both lines belong to the same morphospecies.

To quantitatively characterize the penetrance and consistency of the BS phenotype, we tested more than 100 flies per line (BS and control), all 10-day-old adult males, across five independent trials performed on different days. None of the control flies displayed a bang-sensitive response under identical stimulation. Among BS flies, the stereotyped seizure–paralysis–recovery sequence described above was observed in approximately 90% of individuals tested; the remaining 10% showed an attenuated or ambiguous response.

Video S1: The bang-sensitive (BS) phenotype in *Drosophila funebris*. Following gentle mechanical stimulation (shaking of the culture vial 20 s), flies of the BS line exhibit an initial seizure-like paroxysm lasting ≈15–20 s, characterized by uncoordinated hyperkinetic movements and convulsive leg contractions. This is immediately followed by a motionless paralytic phase of ≈2–2.5 min and subsequent spontaneous recovery. This stereotyped sequence closely mirrors the classical BS phenotype previously described in *D. melanogaster*. The video shows a single, continuous, unedited recording of a representative fly. Control *D. funebris* flies of the same geographic origin show no response to identical stimulation.

### 2.3. Comparative Transcriptomic Analysis of BS and Control Drosophila funebris Isofemale Lines

**Figure 1:**
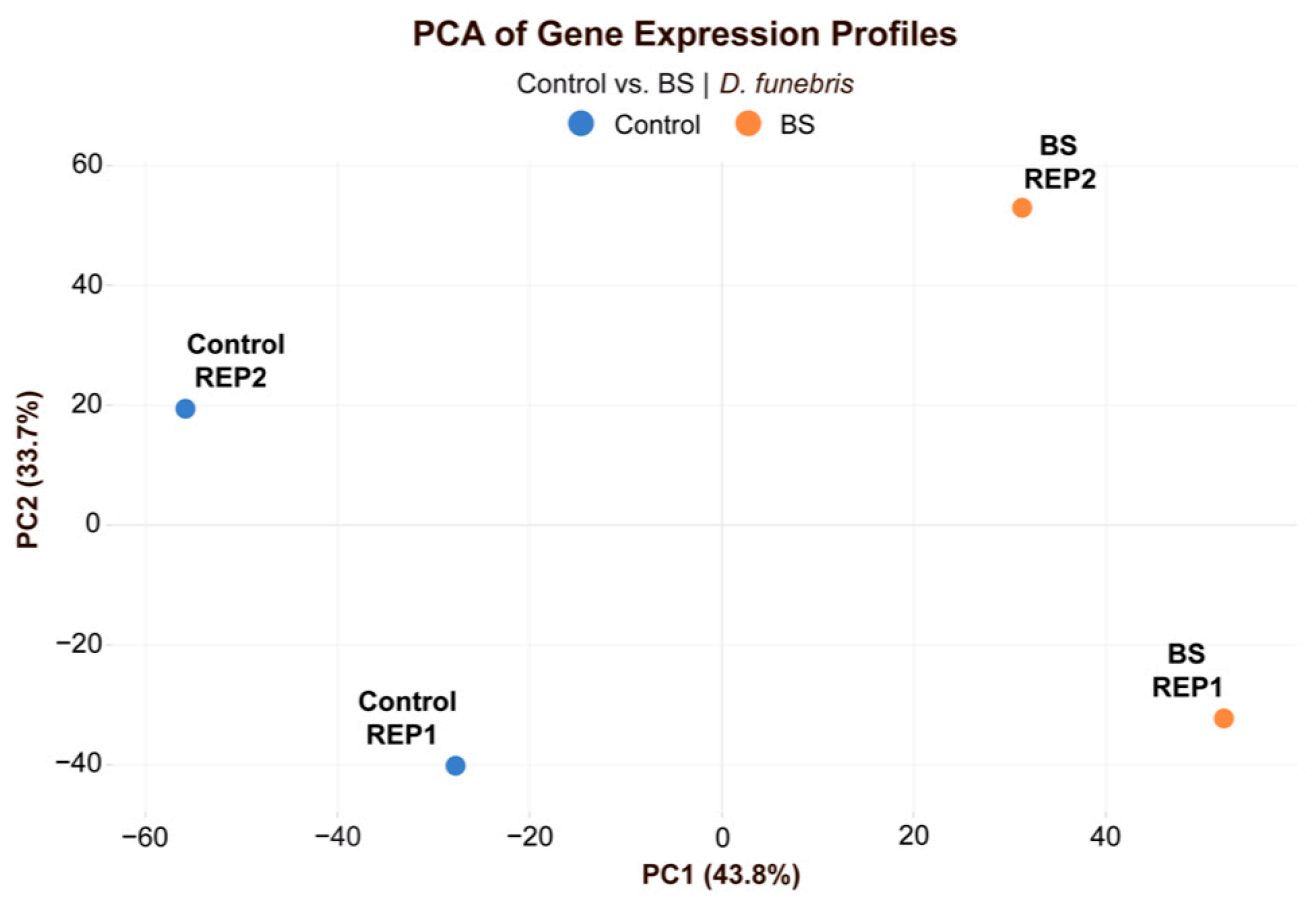
Principal component analysis (PCA) of gene expression profiles in *Drosophila funebris* bang-sensitive (BS) and control strains. Each point represents one RNA-seq sample (control REP1–2 and BS REP1–2). The first principal component (PC1, 43.8% variance) separates the BS and control samples, while the second principal component (PC2, 33.7% variance) captures within-group variability. This indicates robust transcriptomic differences between BS and control flies with good clustering of biological replicates.

Comparative transcriptomic analysis of whole-body lysates from BS and control *Drosophila funebris* lines detected 4336 expressed genes, as summarized in Supplementary Table S2. Principal component analysis (PCA) of variance-stabilized expression values revealed a clear separation between BS and control samples along PC1 (43.8% of the variance), with tight clustering of biological replicates along PC2 (33.7% of the variance) (Figure 1). These results indicate robust global transcriptomic differences between BS and control flies, confirming the suitability of the dataset for differential expression analysis.

**Figure 2:**
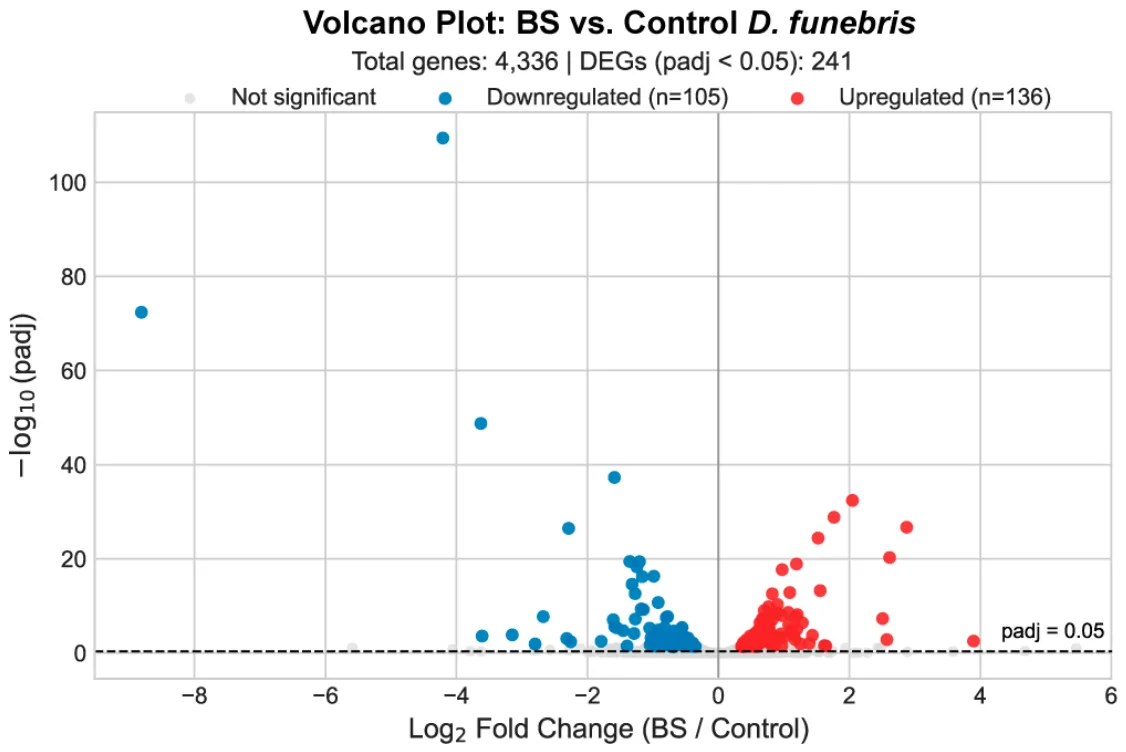
A volcano plot of differentially expressed genes in *Drosophila funebris* bang-sensitive versus controls strains (BS vs. control). Each point represents one gene (*n* = 4336). The x-axis indicates the log_2_fold change (log_2_*FC*), and the y-axis indicates the −log_10_-transformed DESeq2-adjusted *p* value (Benjamini–Hochberg correction). The dashed horizontal line indicates the significance threshold (*p*_adj_ = 0.05). The red points denote significantly upregulated genes in BS (*n* = 136), the blue points denote significantly downregulated genes (*n* = 105), and the gray points denote non-significant genes (*n* = 4095).

Among the 4336 expressed genes, 241 showed statistically significant differential expression (FDR-adjusted *p* < 0.05; Supplementary Table S2). Of these differentially expressed genes (DEGs), 136 were significantly upregulated and 105 were significantly downregulated in the BS line relative to the control. The magnitude of differential expression ranged from −8.81 to +3.90 log_2_fold change (Supplementary Table S2). The volcano plot reveals an asymmetric distribution of DEGs: although upregulated genes outnumber downregulated ones (136 vs. 105), the most statistically significant changes are concentrated among the downregulated transcripts (Figure 2). The majority of expressed genes cluster around log_2_*FC* ≈ 0 with high adjusted *p* values, indicating globally stable transcript levels between BS and control flies, whereas a restricted subset of transcripts displays robust and statistically significant changes in expression (Figure 2; Supplementary Table S2). This pattern suggests that the *D. funebris* bang-sensitive phenotype is associated with targeted transcriptional reprogramming rather than a broad, unspecific perturbation of the transcriptome.

**Figure 3:**
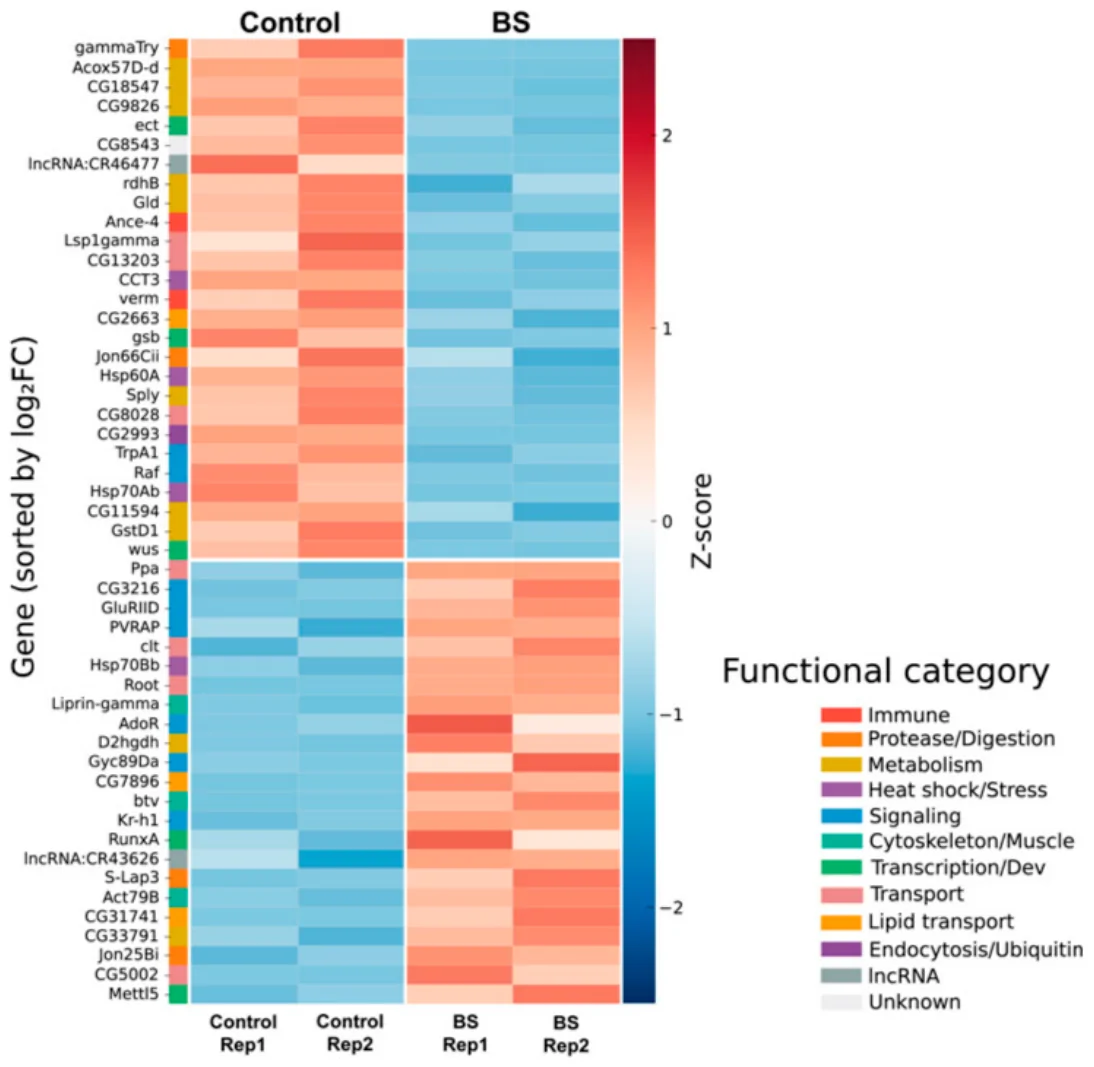
A heatmap of the top 50 differentially expressed genes between *Drosophila funebris* bang-sensitive (BS) and control strains. The rows correspond to individual genes sorted by log_2_fold change, and the columns correspond to RNA-seq samples (control Rep1–2 and BS Rep1–2). The colors represent gene-wise Z-scores of variance-stabilized expression values, with red indicating higher relative expression and blue indicating lower relative expression. The dashed horizontal line separates downregulated (above) from upregulated (below) genes in the BS strain. The colored bar on the left indicates manually assigned functional categories based on FlyBase gene annotations. Full differential expression statistics are provided in Supplementary Table S2.

To explore coordinated transcriptional changes underlying the BS phenotype, we visualized the top 50 differentially expressed genes (ranked by absolute log_2_fold change among DEGs with *p*_adj_ < 0.05; Supplementary Table S2) using a heatmap of gene-wise Z-scores (Figure 3). The heatmap reveals two major clusters of transcripts defined by reciprocal expression patterns between the control and BS strains. Among the downregulated genes, the most prominent functional groups include lipid metabolism (*Acox57D-d*, *Gld*, *Sply*), cellular stress response and protein folding (*Hsp70Ab*, *Hsp60A*, *CCT3*), detoxification (*GstD1*), and transcriptional regulation (*gsb*, *Raf*). The decrease in GstD1 (−1.16) is of particular interest, since Keap1 (+0.48), which retains the antioxidant master regulator CncC (the *Drosophila* orthologue of Nrf2) in the cytoplasm, was upregulated in the same samples, and GstD1 itself belongs to the canonical CncC targets. Combined, these two changes indicate suppression of the CncC-dependent antioxidant program rather than loss of a single detoxifying enzyme. Downregulation of the thermosensitive ion channel *TrpA1* further indicates potential alterations in sensory signal transduction pathways. Conversely, among the upregulated genes, cytoskeletal components (Act79B), transcriptional regulators (RunxA, Kr-h1), and genes involved in metabolic activation (D2hgdh, discussed in detail below; CG3216) were prominently represented. The diversity of affected functional categories suggests that the bang-sensitive phenotype in *D. funebris* involves systemic, coordinated transcriptional changes spanning multiple biological processes.

Gene set enrichment analysis was performed using PANGEA (PAthway, Network and Gene-set Enrichment Analysis) on the FlyRNAi.org platform [36,37]. The background gene set comprised all 4336 genes detected in the RNA-seq experiment, and the query set consisted of the 241 identified DEGs (Supplementary Table S2). Statistical over-representation of GO Biological Process terms was assessed using Fisher’s exact test with Bonferroni, Benjamini–Hochberg, and Benjamini–Yekutieli corrections; terms reaching an adjusted *p* < 0.05 under all three corrections were considered statistically significant (Supplementary Table S3).

**Figure 4:**
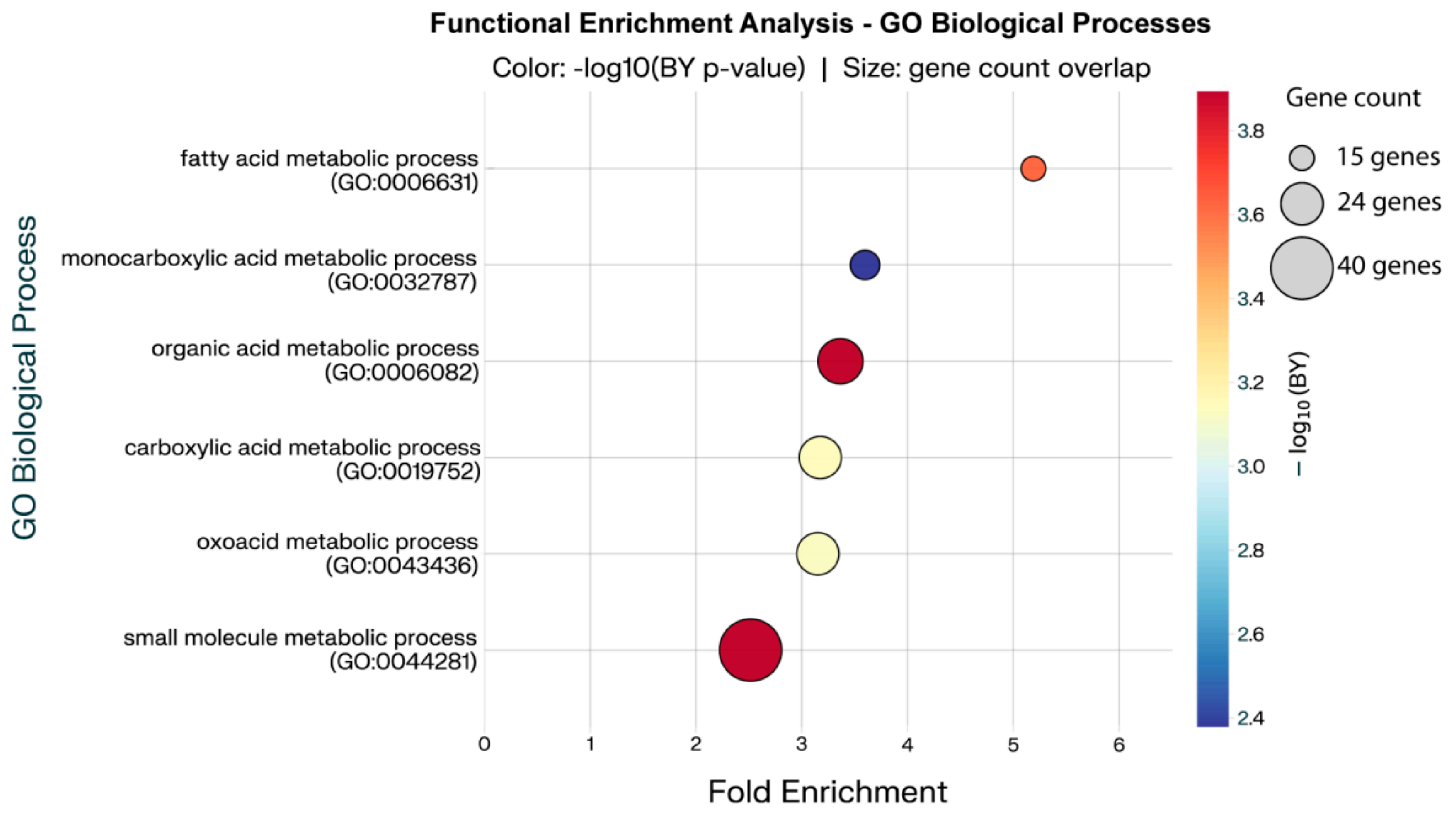
Gene Ontology (GO) Biological Process enrichment analysis of differentially expressed genes in a bang-sensitive strain of *Drosophila funebris*. A bubble plot showing GO terms significantly over-represented among 241 differentially expressed genes (DESeq2, adj. *p* < 0.05) relative to a background of 4336 genes detected by RNA-seq. GO term identifiers are indicated in parentheses next to each term name. Only terms reaching adjusted *p* < 0.05 under all three multiple-testing corrections (Bonferroni, Benjamini–Hochberg, and Benjamini–Yekutieli) are shown. The bubble color indicates the −log_10_-transformed Benjamini–Yekutieli-adjusted *p* value; bubble size indicates the number of query genes overlapping with the GO term. Analysis was performed using PANGEA on the FlyRNAi.org platform [36,37]. Full enrichment results are provided in Supplementary Table S3.

Among the significantly enriched GO Biological Process terms, “fatty acid metabolic process” showed the highest fold enrichment (FE = 5.19, *n* = 15 genes), indicating a pronounced perturbation of lipid metabolism. The remaining enriched terms—“monocarboxylic acid metabolic process” (FE = 3.60), “organic acid metabolic process” (FE = 3.37), “carboxylic acid metabolic process” (FE = 3.18), “oxoacid metabolic process” (FE = 3.15), and “small molecule metabolic process” (FE = 2.52)—reflect a broader disruption of intermediate cellular metabolism linked to the bang-sensitive phenotype in *D. funebris* (Figure 4).

Within the 15 genes contributing to the enrichment of the “fatty acid metabolic process” term, distinct functional subgroups were identified. Genes encoding key enzymes of *de novo* lipogenesis—*FASN1*, *FASN2*, *ACC*, and *ATPCL*—were upregulated, indicating an activation of fatty acid synthesis. In contrast, genes involved in peroxisomal β-oxidation—*Acox57D-d* and *Acsf2*—were downregulated, suggesting impaired fatty acid degradation. Notably, within this metabolic pathway, the peroxisomal acyl-CoA oxidase gene *Acox57D-d* exhibited the most statistically significant and robust downregulation among all lipid-related transcripts (log_2_FC = −4.21, adj. *p* = 4.1 × 10^−110^), signaling a near-complete loss of its transcriptional activity in the mutant line.

Beyond the top 50 genes visualized in Figure 3, inspection of the full DEG set (Supplementary Table S2) identified additional significant changes in energy-metabolism-related genes, including the upregulation of both phosphoenolpyruvate carboxykinases (*Pepck1*, log_2_*FC* = +0.76, adjusted *p* = 1.9 × 10^−6^; *Pepck2*, log_2_*FC* = +0.46, adjusted *p* = 9.0 × 10^−3^) and the insulin-pathway antagonist *ImpL2* (log_2_*FC* = +0.74, adjusted *p* = 2.2 × 10^−7^). *Foxo* (log_2_*FC* = +0.41, adjusted *p* = 0.064) and *AkhR* (log_2_*FC* = +0.40, adjusted *p* = 0.061) showed concordant, but non-significant, directional changes and are not considered DEGs in the present analysis. In *Drosophila*, PEPCK contributes not only to gluconeogenesis but also to glyceroneogenesis, a pathway that generates glycerol-3-phosphate for triacylglycerol synthesis and fatty acid re-esterification [38,39]. The increased expression of both *Pepck1* and *Pepck2* may therefore be consistent with altered glycerolipid metabolism in the studied BS line; however, this interpretation remains hypothesis-generating.

Among the differentially expressed genes, D2hgdh (+1.28), encoding D-2-hydroxyglutarate dehydrogenase, was one of the most strongly upregulated transcripts in the dataset. This enzyme degrades D-2-hydroxyglutarate, a metabolite whose accumulation in humans causes D-2-hydroxyglutaric aciduria, a neurometabolic disorder frequently presenting with epileptic encephalopathy and seizures [40]. Notably, recent functional work in a mammalian model has directly linked reduced D2HGDH activity to increased seizure susceptibility, acting through GSH/Prdx6-mediated oxidative stress in neurons [41]. The marked upregulation of D2hgdh in BS heads may therefore represent a compensatory response aimed at limiting D-2-hydroxyglutarate accumulation and associated oxidative damage, complementing the antioxidant and energy-metabolism shifts discussed above; however, this interpretation remains speculative, as D-2-hydroxyglutarate levels were not measured directly in this study.

As outlined in the Introduction, BS loci can be functionally distinguished as primary drivers or genetic modifiers of seizure susceptibility. The transcriptional profile of whole-body BS *D. funebris* strongly aligns with this framework. The marked downregulation of *Acox57D-d*, independently supported by RT-qPCR, identifies this gene as a leading candidate associated with the molecular phenotype of the studied BS line. Its functional connection to peroxisomal fatty acid metabolism makes it a priority for further investigation; however, the present data do not establish reduced enzyme activity, impaired peroxisomal β-oxidation, or a causal role in bang sensitivity. Beyond its unmatched statistical significance, *Acox57D-d* is distinguished by its direct functional connection to peroxisomal β-oxidation and the downstream neurological context of bang sensitivity—a link further supported by the head-specific lipidomic data presented below (Section 2.5). Concurrently, the downregulation of *eas*—encoding ethanolamine kinase and representing a classical primary BS gene in *D. melanogaster* (Supplementary Tables S1 and S2)—together with the broader transcriptional shift favoring fatty acid synthesis and suppressing fatty acid degradation may collectively constitute a synergistic modifier background that lowers the threshold for neuronal seizure activity and paralysis. Synthesizing these findings, the bang-sensitive phenotype in *D. funebris* is fundamentally rooted in a coordinated transcriptional dysregulation of lipid metabolism, with *Acox57D-d* emerging as the key upstream candidate gene underlying this systemic metabolic imbalance.

### 2.4. RT-qPCR Validation of Acox57D-d Gene

Due to the non-model status of the study organism and the inherent limitations of Liftoff-based genome annotation, we focused validation resources on RT-qPCR confirmation of the key differentially expressed gene, *Acox57D-d*, rather than increasing the number of RNA-seq replicates.

Since the bang-sensitive phenotype manifests as a neurological response to mechanical stimulation, and whole-body RNA-seq cannot resolve tissue-specific expression patterns, RT-qPCR was performed separately on dissected heads and bodies. This design enabled us to assess whether the transcriptional reduction detected in whole-body RNA-seq was also evident in head tissue and whether a comparable difference was detectable in bodies.

**Figure 5:**
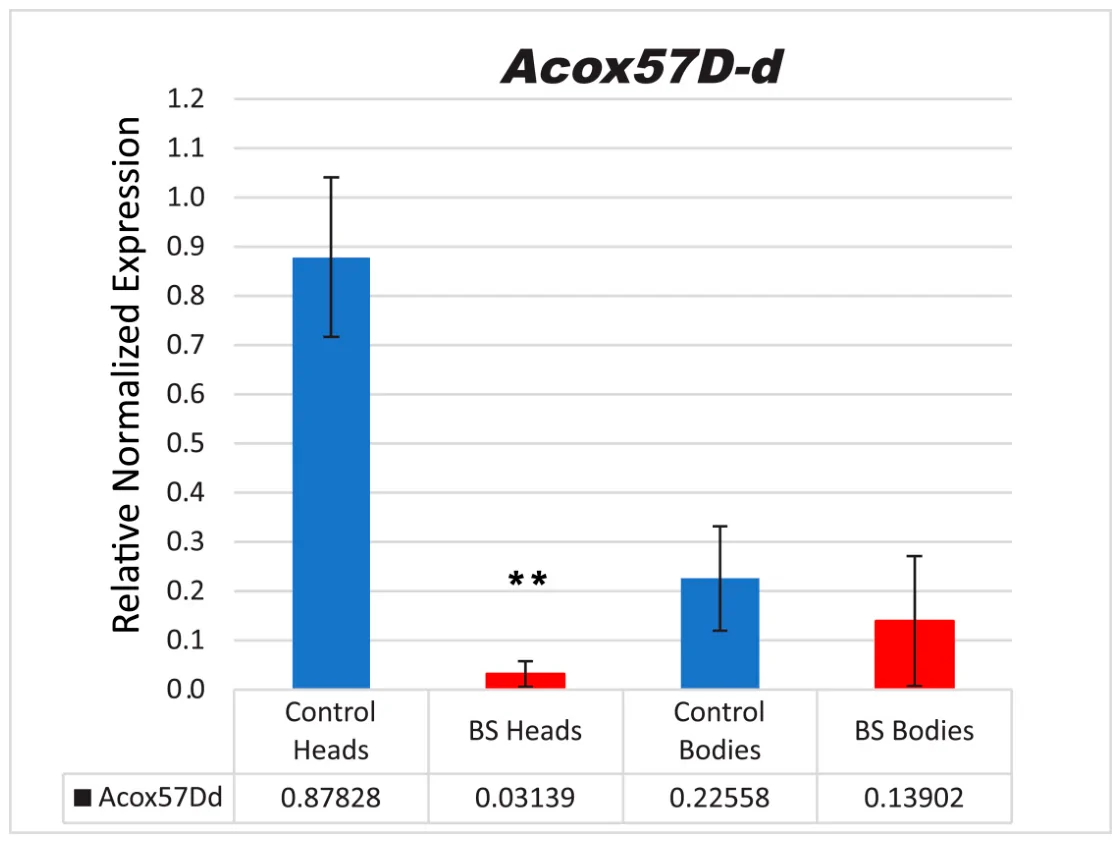
A head-associated reduction in *Acox57D-d* mRNA abundance in bang-sensitive *Drosophila funebris*. Relative normalized *Acox57D-d* mRNA abundance was measured by RT-qPCR in the heads and bodies of control and bang-sensitive (BS) flies and normalized to the geometric mean of the *Tbp* and *LamC* reference genes using an efficiency-corrected comparative approach. The bars represent mean +/− SEM (*n* = 5 independent biological replicates per group; three technical replicates per biological replicate were averaged before normalization and statistical analysis). Shapiro–Wilk tests indicated no significant departure from normality in any group (all *p* > 0.05). Two-sided independent-samples *t*-tests were used for prespecified BS-versus-control comparisons within each tissue. The double asterisk indicates lower expression in BS heads than in control heads (*p* = 0.00211); no statistically significant BS–control difference was detected in the bodies (*p* > 0.05). A two-way ANOVA showed a significant genotype × tissue interaction (*F*(1, 16) = 17.964, *p* = 0.00063), indicating that the BS–control difference differed between heads and bodies.

Shapiro–Wilk tests were performed separately for the four genotype-by-tissue groups (control heads, BS heads, control bodies, and BS bodies; *n* = 5 independent biological replicates per group). No group showed a statistically significant departure from normality (all *p* > 0.05). Prespecified comparisons between control and BS flies were then performed separately within each tissue compartment using two-sided independent-samples *t*-tests. *Acox57D-d* relative mRNA abundance was approximately 28-fold lower in BS heads than in control heads (*p* = 0.00211), whereas no statistically significant difference was detected between BS and control body samples (*p* > 0.05; Figure 5).

Thus, the marked reduction in *Acox57D-d* expression observed by RT-qPCR was detected in head samples but not in body samples. This result is consistent with the possibility that the whole-body RNA-seq signal is driven largely by changes in the head compartment.

To test whether the BS-associated expression difference varied between tissues, we fitted a two-way ANOVA including genotype, tissue, and their interaction. The genotype × tissue interaction was significant (*F*(1, 16) = 17.964, *p* = 0.00063), indicating that the magnitude of the BS–control difference in *Acox57D-d* expression differed between heads and bodies. This result supports a head-associated reduction in *Acox57D-d* expression rather than a uniform tissue-independent difference. Because the assays were conducted on whole heads, this result does not by itself establish a neural-specific effect.

The stronger reduction in *Acox57D-d* expression in heads than in bodies is consistent with differential regulation of this locus across tissue compartments. This pattern could reflect a cis-regulatory change affecting a head-associated regulatory element, analogous to tissue-specific regulatory mechanisms described for other *Drosophila* genes such as *Cyp6g1* [42], or a trans-acting factor that differentially influences *Acox57D-d* transcription across tissues. These possibilities remain to be tested experimentally.

### 2.5. Lipidomic Profiling of Drosophila funebris Heads

To characterize lipid composition differences associated with the bang-sensitive phenotype, we performed untargeted lipidomic profiling of head tissue from BS and control *D. funebris* adults using LC–Q-TOF-MS in both positive and negative ionization modes, with six biological replicates per group. A total of 294 lipid species spanning 14 lipid classes were detected and quantified across all samples (Supplementary Table S4).

**Figure 6:**
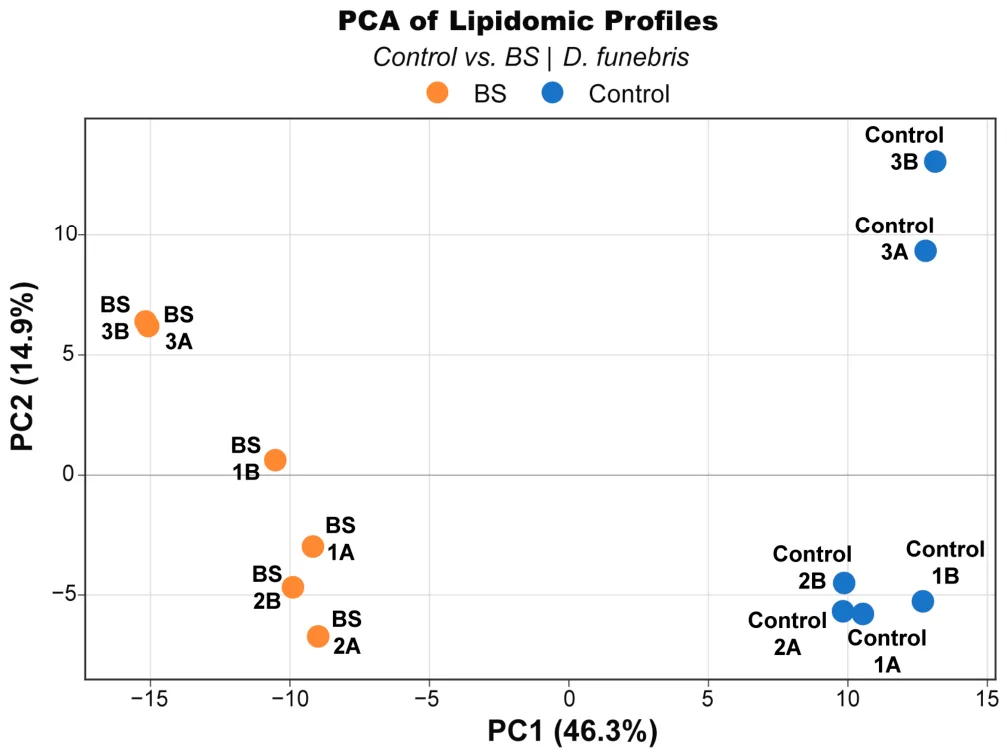
Principal component analysis (PCA) of lipidomic profiles in *Drosophila funebris* adult heads. Each point represents one biological sample (*n* = 6 per group; two aliquots per collection day, three collection days). Bang-sensitive (BS) flies are shown in orange; control flies are shown in blue. PC1 and PC2 explain 46.3% and 14.9% of total variance, respectively.

To assess the overall similarity and reproducibility of samples, we performed principal component analysis (PCA) of normalized lipid abundances (Figure 6). The first two principal components explained 46.3% and 14.9% of the total variance, respectively. PCA revealed a clear separation between BS and control groups along PC1, indicating systematic differences in the global lipid composition between the two phenotypes. Within each group, biological replicates clustered together, confirming the reproducibility of the lipidomic profiles across independent collection days.

**Figure 7:**
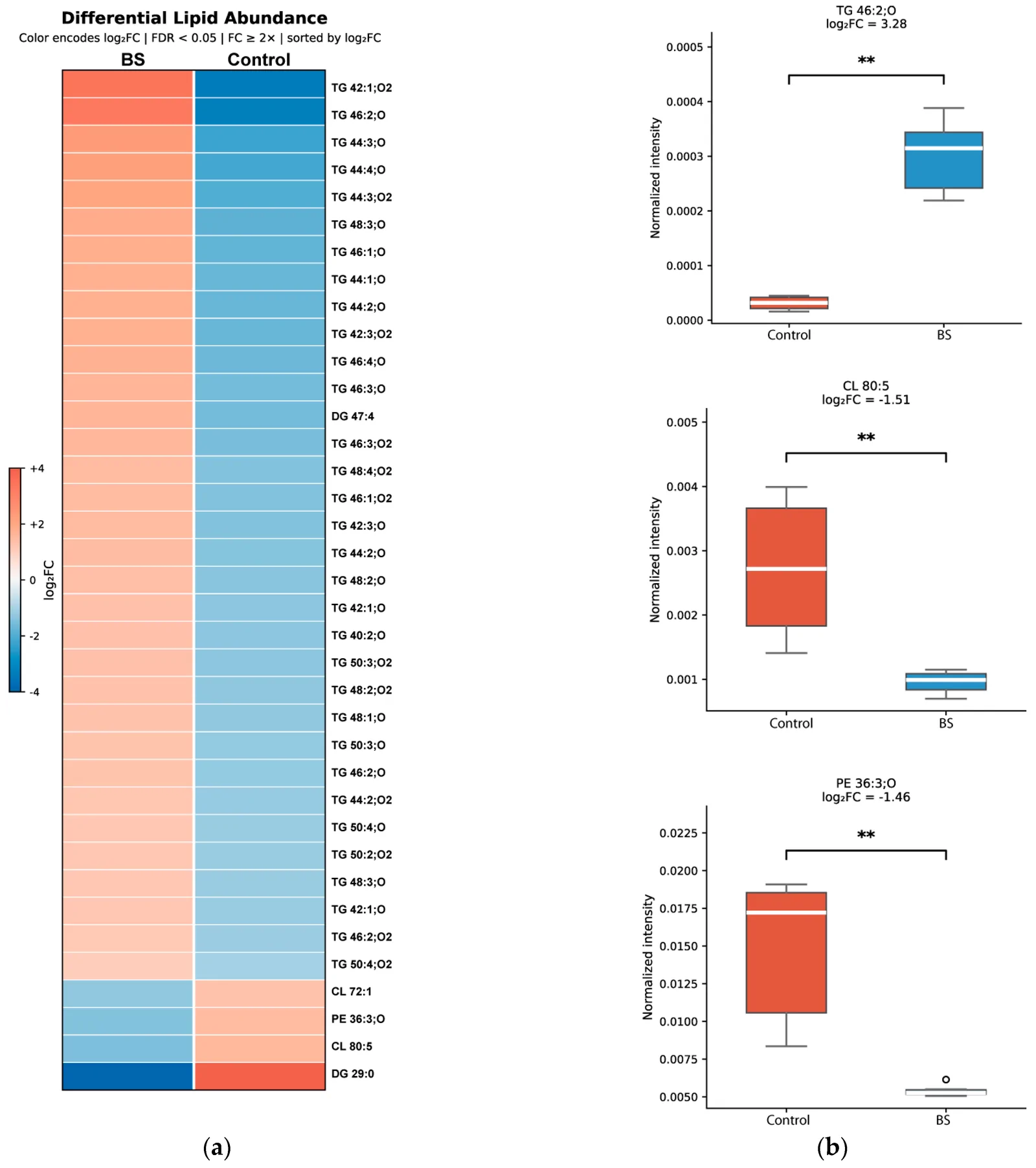
Differential lipid abundance in the heads of bang-sensitive (BS) *Drosophila funebris.* (**a**) A heatmap of significantly altered lipid species between BS and control flies (FDR < 0.05, FC ≥ 2×). Color encodes log_2_ fold change (BS vs. control); red indicates higher abundance in BS flies, blue indicates lower abundance. Lipid species are sorted by log_2_FC. The majority of elevated species are triacylglycerols (TG;O and TG;O2), while reduced species include cardiolipins (CL), phosphatidylethanolamine (PE 36:3;O), and diacylglycerol (DG 29:0). DG 47:4 was elevated in BS heads. (**b**) Box plots illustrating normalized signal intensities for three representative lipid species: TG 46:2;O (log_2_FC = +3.28, elevated in BS), CL 80:5 (log_2_FC = −1.51, reduced in BS), and PE 36:3;O (log_2_FC = −1.46, reduced in BS). The boxes indicate interquartile range; the horizontal line, median; the whiskers, 1.5× IQR; the open circle, outlier. Statistical significance: ** FDR < 0.01.

Statistical comparison between BS and control groups using the two-sided Mann–Whitney U test with Benjamini–Hochberg correction for multiple comparisons revealed 145 lipid species showing statistically significant differences in abundance (FDR < 0.05), spanning 11 lipid classes (Supplementary Table S4). A heatmap visualization of the 37 most differentially abundant lipid species that met both FDR < 0.05 and FC ≥ 2× (Figure 7) revealed a striking dominance of triacylglycerols (TGs) among upregulated species in BS flies. Of the 33 lipids elevated in BS flies, 32 were TGs of varying chain length and degree of unsaturation, predominantly containing 14:0, 16:0, 16:1, and 18:1 fatty acid moieties. One diacylglycerol species (DG 47:4) was also elevated in BS flies. In contrast, the four lipids showing higher abundance in control flies comprised two cardiolipins (CL 72:1 and CL 80:5), one phosphatidylethanolamine (PE 36:3;O), and one diacylglycerol (DG 29:0). A functional summary of these 37 lipid species, including their lipid class, direction of change, and proposed biological relevance, is provided in Supplementary Table S4, Sheet 2.

## 3. Discussion

This study presents the first molecular-genetic and lipidomic characterization of a bang-sensitive strain arising spontaneously in a natural population of *Drosophila funebris*. The central finding is that the BS phenotype observed in the studied BS line is associated with a profound and coordinated dysregulation of lipid metabolism—most prominently, near-complete transcriptional silencing of *Acox57D-d*, the peroxisomal acyl-CoA oxidase, and a corresponding accumulation of oxygenated triacylglycerols in head tissue. This convergence of transcriptomic and lipidomic evidence on a single metabolic axis is striking, and stands in contrast to the diversity of primary BS loci previously characterized in *D. melanogaster* (Supplementary Table S1), none of which have been linked to peroxisomal β-oxidation. Below, we discuss the functional implications of *Acox57D-d* downregulation for neuronal excitability, the significance of the lipidomic phenotype in the context of known BS biology, and the broader relevance of these findings for the comparative genetics of seizure-and-paralysis susceptibility.

The accumulation of TG can be considered a direct consequence of fatty acid buildup in the cell cytoplasm, resulting from the block of peroxisomal fatty acid β-oxidation caused by low expression of the *Acox57D-d* gene in the heads of bang-sensitive flies. The cell neutralizes these biologically active fatty acids by converting them into neutral triglycerides [43,44]. The pronounced accumulation of TG species in BS heads—predominantly oxygenated forms carrying one or two oxygen modifications (TG;O and TG;O2)—is consistent with an increase in neutral lipid storage, potentially in the form of lipid droplets, which have been previously associated with neuronal stress responses and neurodegeneration in *Drosophila* models [45].

Beyond the accumulation of neutral lipids, we also observed changes in phospholipid classes essential for mitochondrial membrane integrity. The simultaneous reduction in cardiolipin species (CL 72:1 and CL 80:5) and oxygenated phosphatidylethanolamine (PE 36:3;O) in BS fly heads is consistent with a broader impairment of mitochondrial membrane phospholipid homeostasis. Both lipid classes are essential structural and functional components of the inner mitochondrial membrane: cardiolipin stabilizes respiratory chain supercomplexes and is required for cytochrome c oxidase activity, while PE is synthesized in part directly within mitochondria via the phosphatidylserine decarboxylase (PSD) pathway and is critical for mitochondrial respiratory capacity, morphology, and distribution [46,47]. In *Drosophila* neurons, loss of PE biosynthesis causes defective phototransduction and light-independent neurodegeneration, demonstrating that PE homeostasis is required for neuronal function and integrity [47]. Thus, the coordinated depletion of CL and PE species suggests that mitochondrial membrane dysfunction may be a contributing factor to the neurological phenotype of bang-sensitive *D. funebris*.

We hypothesize that the reduction in CL and PE levels in the heads of bang-sensitive flies may be linked, at least in part, to oxidative stress arising from VLCFA accumulation in the cytoplasm. It is known that blockade of peroxisomal β-oxidation leads to VLCFA accumulation both within peroxisomes [48,49]—compromising their capacity to detoxify H_2_O_2_—and in the cytoplasm, where it can activate compensatory CYP450-mediated ω-oxidation [50,51,52]. Partial entry of VLCFAs/acylcarnitines into mitochondria may further overload the respiratory chain and increase electron leakage to oxygen (superoxide) [53,54], collectively contributing to elevated ROS levels in the cell, particularly within mitochondria. One plausible explanation is that the observed decrease in CL and PE reflects oxidative modification of these phospholipids in mitochondria; however, we note that the oxidized forms of these lipids are highly unstable and therefore difficult to detect with the LC–Q-TOF-MS approach used here, which may explain why direct evidence for their oxidized derivatives was not observed in our dataset.

Diacylglycerols play a central role at the intersection of structural lipid synthesis and lipid second messenger signaling [55,56]. DG 29:0 and DG 47:4 showed opposing changes in abundance, which may reflect a remodeling of the diacylglycerol pool with potential downstream effects on membrane organization. DG 29:0, as a saturated medium-chain lipid, may contribute to the density and geometric packing required for lipid raft integrity [57,58], whereas DG 47:4, with its four double bonds, could plausibly destabilize the tightly packed raft environment given its bulkier, more disordered acyl chain structure [59,60]. Synaptic Na^+^/K^+^-ATPases are known to be preferentially localized within lipid rafts—dense membrane microdomains that help position the pump in proximity to voltage-gated channels. We therefore speculate that the shift in the relative abundance of DG 29:0 and DG 47:4 could influence local membrane packing and, potentially, the functional efficiency of synaptic Na^+^/K^+^-ATPases, although this link remains to be experimentally tested.

The most parsimonious explanation for the BS phenotype in *D. funebris* centers on the near-complete transcriptional silencing of *Acox57D-d*, encoding the rate-limiting enzyme of peroxisomal fatty acid β-oxidation. Peroxisomal β-oxidation is the primary catabolic route for very-long-chain and branched-chain fatty acids, as well as for oxygenated fatty acid derivatives; its impairment leads to the accumulation of these substrates, which are subsequently re-esterified into neutral lipids [61,62]. This is precisely the pattern observed in the lipidomic profile of BS *D. funebris* heads, where oxygenated triacylglycerol species predominate among elevated lipids—a finding that provides independent, biochemical validation of the transcriptional deficit in *Acox57D-d*.

The head-specific downregulation of *Acox57D-d*, with no corresponding change in body tissue, strongly suggests that impaired peroxisomal β-oxidation is a head-restricted phenomenon in bang-sensitive *D. funebris*. This tissue specificity is consistent with the known role of peroxisomal β-oxidation in neuronal membrane homeostasis and very-long-chain fatty acid metabolism in the nervous system [63,64], and argues against a systemic metabolic disturbance as the primary driver of the phenotype. Instead, it supports the hypothesis that *Acox57D-d* suppression acts locally within neural tissue to alter the lipid environment of neurons—a conclusion further reinforced by the head-specific accumulation of oxygenated TG species in the lipidomic data.

Two caveats temper this neural-specific interpretation. First, according to FlyAtlas2 tissue expression data for *D. melanogaster* [65], genes of the fatty acid β-oxidation pathway—including *Acox57D-d*—are consistently under-represented in isolated brain/CNS tissue (enrichment scores of 0.1–0.2 relative to whole body) compared with more metabolically active tissues such as the midgut, consistent with the brain’s general reliance on glucose rather than direct fatty acid oxidation for energy metabolism. Notably, *Acox57D-d* itself shows nearly 5-fold higher expression in whole head samples than in isolated brain/CNS tissue (FPKM 1.4 vs. 0.3), indicating that a substantial portion of head-associated *Acox57D-d* transcript may originate from non-neural head tissue. This tissue-compositional heterogeneity is further corroborated at the lipidomic level: comparative MS profiling of whole head versus dissected brain in *D. melanogaster* has shown that these two tissue types differ substantially in overall lipid class composition (e.g., TG-dominant in whole head versus PC-dominant in isolated brain), consistent with a non-negligible contribution of non-neural head structures to bulk head lipidomic and transcriptomic signals [66]. This underscores the importance of confirming, in future work, whether the pronounced downregulation observed in BS *D. funebris* heads reflects a genuinely neural-specific regulatory lesion or a broader disruption affecting non-neural components of the head. Second, cis-regulatory elements controlling tissue- and context-specific gene expression can evolve independently of coding sequences. Consequently, genes participating in the same metabolic pathway may be regulated by distinct promoters and enhancers. It is therefore possible that the reduced head expression of *Acox57D-d* reflects a change in a regulatory element acting locally at this locus, rather than a mechanism affecting fatty acid β-oxidation genes more broadly. This distinction could be examined in future studies using allele-specific expression analysis, promoter/enhancer sequencing, or chromatin-accessibility profiling.

The functional link between peroxisomal dysfunction and neuronal hyperexcitability is not yet fully established, but several non-exclusive pathways merit consideration. Accumulation of very-long-chain fatty acids (VLCFAs) and their oxygenated derivatives alters the biophysical properties of neuronal membranes, directly affecting ion channel gating and excitability. In parallel, peroxisomal dysfunction drives secondary mitochondrial impairment, reflecting the well-established peroxisome–mitochondria crosstalk in fatty acid β-oxidation. Reduced *Acox57D-d* activity is expected to block peroxisomal β-oxidation and cause accumulation of unmetabolized, potentially peroxidized fatty acid intermediates, which are diverted into triacylglycerol storage—in agreement with the elevated TG levels observed in BS heads.

This lipotoxic and oxidative burden likely extends to mitochondrial membranes. Because cardiolipin (CL) and phosphatidylethanolamine (PE) are the principal unsaturation-rich, non-bilayer phospholipids of the inner membrane, they are preferentially susceptible to peroxidative degradation. This aligns with the concurrent depletion of CL and PE species observed in BS heads, which we interpret as indicative of compromised inner mitochondrial membrane integrity. Specifically, VLCFA accumulation resulting from *Acox57D-d* downregulation may perturb peroxisomal redox balance while activating compensatory cytochrome P450-mediated ω-oxidation in the cytoplasm; partial mitochondrial entry of unmetabolized VLCFAs or acylcarnitines may further increase electron leakage and superoxide production, collectively elevating cellular ROS.

We note, however, that the oxidized derivatives of CL and PE predicted by this model were not directly detected in our dataset, likely reflecting their inherent chemical instability and poor recovery by the LC–Q-TOF-MS workflow used here; this analytical limitation is addressed directly in the planned TBARS and DCFH-DA validation experiments outlined below. Because both lipids are required for respiratory chain supercomplex assembly and maintenance of the mitochondrial membrane potential, their loss may further impair oxidative metabolism, establishing a self-reinforcing cycle of lipid accumulation and mitochondrial dysfunction that could contribute to the energy deficits underlying seizure-like activity [67,68,69,70,71,72,73].

The contrasting shifts between the downregulated DG 29:0 and upregulated DG 47:4 species point to two distinct, independently arising facets of the metabolic disruption in the bang-sensitive line, rather than a single unified process. The accumulation of the ultra-long-chain DG 47:4 plausibly reflects a host-intrinsic bottleneck: very-long-chain fatty acids (VLCFAs, ≥C22) are normally channeled into peroxisomes for β-oxidation, and head-specific silencing of *Acox57D-d* may divert unmetabolized VLCFAs into neutral glycerolipid storage—directly linking this change to the primary lesion identified in this study.

The marked reduction in DG 29:0 is an observed lipidomic difference between BS and control heads. This lipid is not a direct expected substrate of *Acox57D-d*-dependent peroxisomal β-oxidation; therefore, the present data do not identify a mechanism responsible for its depletion. As a speculative possibility based on the literature, altered microbiota-dependent odd-chain fatty acid metabolism could contribute to this signature, but this hypothesis was not tested in the present study.

Two non-exclusive routes could, in principle, link microbiota composition to host DG metabolism: propionate-primed biosynthesis of short- and medium-chain OCFAs, for which propionic acid has been identified as a dietary precursor of OCFA-containing phospholipids in *Drosophila* [74]; and a microbial acetate-dependent route. In *Drosophila*, *Acetobacter pomorum*-derived acetate can modulate host insulin/IGF and enteroendocrine IMD/Tachykinin signaling, thereby influencing lipid homeostasis [75,76,77]. BS flies also showed coordinated increased expression of SREBP and several lipogenic genes, including *AcCoAS*, *ATPCL*, *ACC*, *FASN1*, and *FASN2*, together with a substantial reduction in expression of *gammaTry*, a gene annotated as encoding an intestinal trypsin-like protease in *D. melanogaster* (log_2_*FC* = −8.81, *p*_adj_ = 4.1 × 10^−73^; Supplementary Table S2). Because intestinal protease expression in *Drosophila* can be influenced by nutritional state, intestinal physiology, and host–microbiota interactions, the reduced expression of *gammaTry* raises the possibility of an additional non-neural contribution to the BS-associated transcriptional profile. These observations are consistent with, but do not establish, a causal link between microbiota composition, altered host lipid metabolism, and the specific depletion of DG 29:0; nor do they establish a link to *Acox57D-d* deficiency. The selective reduction in this single odd-chain DG species relative to other DG species in our dataset remains unexplained, and chain-level resolution of its acyl composition was not obtained. We therefore raise this possibility only as a direction for future investigation.

Genetically characterized bang-sensitive mutants of *D. melanogaster* have been grouped into two distinct functional categories (Supplementary Table S1): (1) those inducing a mitochondrial bioenergetic crisis via disrupted ATP production or transport (e.g., *tko*, *sesB*, *eas*), and (2) those directly impairing ion transport and plasma membrane excitability (e.g., *para*, *ATPα*, *jus*). Remarkably, our natural BS strain of *D. funebris* demonstrates that a single locus, *Acox57D-d*, can bridge these two independent pathological pathways through tissue-specific membrane lipid remodeling. On one hand, silencing of peroxisomal β-oxidation triggers an oxidative cascade that depletes critical structural phospholipids of the inner mitochondrial membrane—cardiolipin and phosphatidylethanolamine—mirroring the energetic-starvation profile of the mitochondrial BS flies. On the other hand, it induces a divergent diacylglycerol shift (depletion of dense DG 29:0 and accumulation of more fluid DG 47:4). This dual effect may simultaneously limit the metabolic substrate (ATP) available to the Na^+^/K^+^-ATPase and alter its lipid microenvironment within synaptic lipid rafts, echoing aspects of the transport failure seen in *para*, *jus*, and *ATPα* mutants. Thus, *Acox57D-d* emerges as a candidate node linking metabolic and biophysical mechanisms of seizure and paralysis within this species.

Whether these lipidomic changes influence mitochondrial function, ATP availability, membrane biophysical properties, Na^+^/K^+^-ATPase activity, or neuronal excitability remains unknown. The present data do not directly assess peroxisomal β-oxidation, oxidative stress, mitochondrial function, membrane organization, or ion-transport activity. Thus, *Acox57D-d* should be regarded as a leading candidate gene associated with the BS phenotype and the observed lipidomic signature, rather than as a demonstrated link between metabolic and membrane-based pathways. Functional genetic and biochemical experiments will be required to test this model.

It is noteworthy that *Acox57D-d* has not previously been identified as a BS locus in *D. melanogaster*, where the characterized BS genes (Supplementary Table S1) primarily affect ion channel and mitochondrial function. The present findings therefore expand the molecular landscape of bang sensitivity to include peroxisomal lipid catabolism. Notably, single-enzyme defects of peroxisomal β-oxidation in humans, including ACOX1 deficiency, are associated with progressive neurodegeneration and seizure activity, suggesting that the link between impaired peroxisomal fatty acid oxidation and neuronal hyperexcitability may be phylogenetically conserved and extend beyond *Drosophila* models. In this light, the BS *D. funebris* strain described here may provide a useful experimental system for investigating peroxisomal β-oxidation-related epilepsies, including ACOX1 deficiency, by enabling genetic and pharmacological manipulation of a naturally occurring, peroxisome-linked BS phenotype. While further work is required to establish the extent to which *Acox57D-d* dysfunction in *D. funebris* recapitulates the cellular pathology of human ACOX1-associated peroxisomal acyl-CoA oxidase deficiency (human *ACOX1* being the ortholog of *Drosophila Acox57D-d*), the combination of a clearly defined genetic lesion, a robust and quantifiable seizure phenotype, and an accessible lipidomic readout makes this system a promising platform for mechanistic studies and preclinical screening of candidate therapeutics targeting this class of metabolic epilepsies [78,79]. Although single-enzyme defects of peroxisomal β-oxidation, including human *ACOX1* deficiency, can involve neurological manifestations and seizures, the present data do not establish that the *D. funebris* BS line models *ACOX1*-related disease. Rather, this naturally occurring BS line may provide a potentially useful comparative system for investigating relationships between altered lipid metabolism and mechanically induced seizure-like/paralytic susceptibility. Functional genetic and biochemical studies will be required to determine the relevance of this system to specific disease mechanisms.

The behavioral repertoire of the BS *D. funebris* strain—mechanical stimulation triggering a rapid transition into a motionless state followed by spontaneous recovery—invites comparison with thanatosis (death-feigning), a widely conserved reflex of tonic immobility in insects typically elicited by mechanical disturbance or predator attack. The existence of a naturally occurring BS phenotype in a wild *D. funebris* population raises the intriguing possibility that seizure-susceptibility alleles can be maintained in nature because, at subthreshold levels of expression or under specific environmental conditions, they mimic or enhance thanatosis-like behaviors that confer a survival advantage—a notion consistent with the recently proposed “upside of epilepsy” hypothesis [29]. In this context, BS *D. funebris* provides a unique opportunity to examine how a seizure-prone nervous system behaves in ecologically relevant settings, and to test whether bang-sensitive flies show altered thresholds or dynamics of thanatosis-like responses compared with non-BS conspecifics. Such studies may help bridge the gap between laboratory models of epilepsy and the adaptive behavioral repertoire of insects in the wild, and clarify whether seizure-susceptibility alleles can be co-opted by natural selection as part of an antipredator strategy rather than being purely deleterious.

## 4. Limitations and Future Directions

Several limitations should be considered when interpreting the present findings.

First, the initial whole-body RNA-seq comparison included only two biological replicates per condition. This limited replication constrains the reliability of transcriptome-wide differential expression analysis and pathway-level enrichment, particularly for genes with modest fold changes. Accordingly, the complete set of 241 differentially expressed genes and the associated functional-enrichment results should be considered exploratory and hypothesis-generating. The large reduction in *Acox57D-d* expression (log_2_*FC* = −4.21, adjusted *p* = 4.1 × 10^−10^) is supported more strongly because it was independently confirmed by RT-qPCR in a separate set of five biological replicates per group. Although large-effect genes may be detected more reliably than genes with modest fold changes in low-replication RNA-seq experiments [80], two biological replicates per condition are not generally sufficient for definitive transcriptome-wide conclusions. Independent validation and additional replication will be required to assess the robustness of the remaining DEG set and the broader pathway-level interpretation.

Second, although the convergence of RNA-seq, RT-qPCR, and lipidomic findings is consistent with a possible relationship between reduced *Acox57D-d* expression, altered lipid metabolism, and seizure susceptibility, direct functional evidence is required to test this hypothesis. Most importantly, genetic rescue of *Acox57D-d* expression in the BS background and, conversely, targeted tissue-specific suppression of *Acox57D-d* in a non-BS strain will be required to determine whether reduced *Acox57D-d* expression contributes causally to bang sensitivity. Such gain- and loss-of-function experiments are standard approaches for testing causality in *Drosophila* seizure models and would also help clarify whether the observed head-associated reduction in *Acox57D-d* expression reflects a cis-regulatory change at this locus or a broader trans-acting regulatory mechanism. The present study did not measure *Acox57D-d* enzymatic activity, peroxisomal β-oxidation flux, VLCFA abundance, reactive oxygen species, mitochondrial function, ATP availability, Na^+^/K^+^-ATPase activity, or neuronal excitability, and it did not identify a DNA sequence variant or other genetic lesion at the *Acox57D-d* locus.

Third, the current study does not fully resolve the precise cellular and organelle-level loci of the observed metabolic disturbances. As noted above, whole head samples likely contain a mixture of neural and non-neural tissue, and *Acox57D-d* itself is more highly expressed in whole heads than in isolated brain/CNS tissue in *D. melanogaster*; more spatially resolved data will therefore be required to distinguish neuronal from glial and non-neural contributions to the observed lipid imbalance, given the extensive neuron–glia coupling in lipid metabolism within the fly nervous system. To address these spatial and structural uncertainties, future work will employ comparative transmission electron microscopy (TEM) of the *D. funebris* brain, allowing direct visualization of potential structural aberrations in synaptic vesicle density, synaptic cleft architecture, and mitochondrial cristae alignment, which we hypothesize may be altered as a consequence of the observed lipid transitions.

Fourth, while our lipidomic data demonstrated a profound depletion of mitochondrial cardiolipin (CL) and phosphatidylethanolamine (PE), the upstream biophysical mechanisms driving this collapse remain unverified. We hypothesize that the lack of peroxisomal processing generates a localized oxidative cascade that degrades these labile structural phospholipids. Future biochemical validation will include quantitative oxidative stress assays, specifically the Thiobarbituric Acid Reactive Substances (TBARS) assay to measure malondialdehyde (MDA) accumulation as a stable byproduct of lipid peroxidation, and the fluorogenic 2′,7′-dichlorodihydrofluorescein diacetate (DCFH-DA) assay to track the kinetics of total reactive oxygen species (ROS) production in fresh brain tissue. Because CL and PE depletion could plausibly contribute to mitochondrial decay and subsequent energy failure, direct brain bioenergetics will also be assessed using luciferase-based bioluminescence ATP assays to quantify ATP availability for the synaptic Na^+^/K^+^-ATPase.

Finally, the systemic and genetic architecture of the BS phenotype in this natural *D. funebris* strain remains unresolved. The present data support *Acox57D-d* as the primary candidate locus, but whole-body transcriptomics also revealed a broader background involving non-neural pathways—most notably the sharp downregulation of the intestinal protease *gammaTry* and the marked depletion of DG 29:0, an odd-chain DG species that prompted the tentative microbiota-related hypothesis discussed above. This raises the possibility of disruption along a gut–brain–microbiota axis, although this hypothesis remains to be directly tested. This possibility is further supported by recent functional evidence from a distinct *Drosophila* seizure model: in flies carrying a gain-of-function mutation in the voltage-gated sodium-channel gene *paralytic* (*para*^Shu^), elimination of commensal gut bacteria significantly reduced seizure-like phenotype severity, whereas mono-association with either *Lactobacillus* or *Acetobacter* species restored and exacerbated seizure susceptibility, apparently through suppression of Nrf2-mediated antioxidant signaling in the gut [81]. Although this mechanism was demonstrated in a genetically distinct BS model and has not been tested here, it suggests that commensal bacteria—including taxa such as *Acetobacter*—may modulate seizure susceptibility in *D. funebris* by influencing host oxidative stress status, thereby complementing the peroxisome-centered mechanism proposed above. Future studies will therefore use 16S rRNA gene amplicon sequencing to characterize microbiome differences in the BS strain and test whether dysbiosis or shifts in specific taxa, including *Lactiplantibacillus plantarum* and *Acetobacter pomorum*, modify seizure susceptibility through systemic acetate- or nutrient-dependent signaling. Combined, these experiments will test whether the phenotype reflects a major-effect neural lesion accompanied by modifier alleles and microbial interactions, rather than a purely monogenic defect.

Because this study compares a single wild-derived bang-sensitive (BS) isofemale line with a single control isofemale line, it remains unknown whether the observed transcriptomic and lipidomic differences are specifically associated with bang sensitivity or partly reflect other genetic differences between these particular lines. Accordingly, it remains to be determined whether the same molecular signature, including reduced *Acox57D-d* expression, recurs in other independently derived BS lineages or whether phenotypically similar BS strains can arise through etiologically distinct mechanisms.

## 5. Conclusions

This study provides a molecular and lipidomic characterization of a naturally occurring bang-sensitive (BS) strain of *Drosophila funebris*. Comparative transcriptomic analysis identified a distinct expression profile associated with the BS phenotype, with fatty acid metabolism emerging as the most strongly enriched functional category. Among the differentially expressed genes, the peroxisomal acyl-CoA oxidase gene *Acox57D-d* showed substantial downregulation, which was independently supported by RT-qPCR analysis of head tissue.

Untargeted lipidomic profiling revealed remodeling of the head lipidome in BS flies, including increased relative abundance of predominantly oxygenated triacylglycerol species (TG;O and TG;O2), reduced relative abundance of selected cardiolipin and phosphatidylethanolamine species, and opposing changes in two diacylglycerol species, with reduced DG 29:0 and increased DG 47:4. Taken together, these transcriptomic and lipidomic findings identify *Acox57D-d* as a leading candidate gene associated with the BS phenotype and highlight altered lipid homeostasis in this strain. Functional genetic and biochemical experiments will be required to determine whether reduced *Acox57D-d* expression contributes causally to bang sensitivity and to define the relevant cellular mechanisms.

## 6. Materials and Methods

### 6.1. Genetic Identification of Drosophila funebris

Genetic identification was performed by comparing nucleotide sequences of extended fragments of the mitochondrial *COI* gene, following the recommendations of the Barcode of Life Data Systems (BOLD) platform (https://www.boldsystems.org, accessed on 2 September 2026), a centralized bioinformatics resource for DNA-based biodiversity analysis [34,35].

DNA was extracted from 20 to 25 flies using standard phenol–chloroform extraction. Polymerase chain reaction (PCR) was performed using the primer pair LepF1/LepR1: 5′-ATTCAACCAATCATAAAGATATTGG-3′/5′-TAAACTTCTGGATGTCCAAAAAATCA-3′ [82]. PCR was carried out on a Primus 25 Thermal Cycler (MWG-Biotech, Ebersberg, Germany) using the Encyclo Plus PCR kit (Evrogen, Russia) according to the manufacturer’s instructions. The amplification protocol consisted of an initial denaturation step at 95 °C for 5 min, followed by 30 cycles of 94 °C for 1 min, 52 °C for 1 min, and 72 °C for 1 min, with a final extension step at 72 °C for 7 min. PCR products were analyzed by electrophoretic separation on a 1% agarose gel in Tris-acetate buffer containing trace amounts of a 10% aqueous ethidium bromide solution.

The amplified DNA fragment was excised from the gel under mild UV illumination and purified using the Cleanup Mini kit (Evrogen, Moscow, Russia) according to the manufacturer’s instructions. Sequencing of the *COI* gene fragments was performed by the Sanger method [83], with subsequent deposition in the BOLD and GenBank databases.

### 6.2. Behavioral Assay for the Bang-Sensitive Phenotype

Individual 10-day-old adult male flies were placed in an empty glass culture vial (without food medium) and subjected to mechanical stimulation by tapping the vial against a rubber mat on the bench surface for approximately 20 s of light, repeated tapping. Flies were scored as bang-sensitive if they displayed the stereotyped seizure-like paroxysm followed by a motionless paralytic phase and subsequent spontaneous recovery, as illustrated in Video S1. Behavioral testing was performed on more than 100 flies per line (BS and control) across five independent trials conducted on different days. Each trial used a distinct set of flies, and no individual was tested more than once.

### 6.3. Transcriptomic Analysis

#### 6.3.1. Library Preparation

Total RNA was extracted from four samples (Control_Rep1, Control_Rep2, BS_Rep1, BS_Rep2), each pooled from 20 ten-day-old males, using the ExtractRNA reagent (Evrogen, Russia) according to the manufacturer’s protocol. To reduce residual genomic DNA, RNA samples were treated with recombinant duplex-specific nuclease (DSN; Evrogen, Russia) according to the manufacturer’s protocol. Total RNA quality was assessed by horizontal gel electrophoresis. Ribosomal RNA depletion and cDNA synthesis were performed using the Illumina TruSeq Stranded Total RNA with Illumina Ribo-Zero Plus rRNA Depletion kit (Illumina, San Diego, CA, USA), according to the manufacturer’s instructions. Given the exploratory nature of this comparison and the well-established robustness of RNA-seq for detecting large-effect differential expression even with limited replication, two biological replicates per group were used, with key candidate genes subsequently validated independently by RT-qPCR.

#### 6.3.2. Sequencing and Preprocessing

Libraries were sequenced on an Illumina NovaSeq X platform to generate 150 bp paired-end reads. FASTQ files were generated using BCL Convert v4.2.4 (Illumina, San Diego, CA, USA). Initial read quality was assessed using FastQC v0.12.1, and summary reports were generated using MultiQC v1.16. Adapter trimming and read length/quality filtering were performed using Trim Galore v0.6.10. Post-trimming sequence quality was reassessed using FastQC v0.12.1. A total of 361,649,400 filtered paired-end reads were retained across the four libraries (Control_Rep1: 94,482,830; Control_Rep2: 85,589,966; BS_Rep1: 97,807,042; BS_Rep2: 83,769,562).

#### 6.3.3. Mapping and Quantification

The *D. funebris* genome assembly (GCA_958295475.1) was used as the reference genome. The *D. melanogaster* reference annotation (Release 6, plus ISO1 MT; GCF_000001215.4) was transferred to the *D. funebris* assembly using Liftoff v1.6.3 [33]. Liftoff was run with default mapping thresholds (minimum sequence identity = 0.5, minimum alignment coverage = 0.5) and the -polish option, which re-aligns and refines CDS boundaries after initial mapping, using the following command:

liftoff -g GCF_000001215.4/genomic.gff -o GCA_958295475.1/genomic_liftoff.gff -u GCA_958295475.1/genomic_liftoff_unmapped.gff -polish -p 64 GCA_958295475.1_idDroFune2.1_genomic.fna GCF_000001215.4_Release_6_plus_ISO1_MT_genomic.fna

Under Liftoff’s default behavior, partial mappings are retained rather than discarded at the mapping stage, allowing coverage and identity to be inspected downstream. Of the 13,981 protein-coding genes annotated in the *D. melanogaster* reference genome, 4643 (33.2%) were successfully transferred to the *D. funebris* assembly as protein-coding genes (median alignment coverage = 0.81, median sequence identity = 0.60; 20.4% flagged as partial_mapping and 24.5% as low_identity by Liftoff). Given this mapping quality profile, individual gene-level annotations transferred by Liftoff should be interpreted with corresponding caution.

Because the central finding of this study depends on correctly distinguishing the peroxisomal acyl-CoA oxidase gene *Acox57D-d* from its tandem paralog *Acox57D-p*, we verified its annotation transfer specifically. *Acox57D-d* mapped at coverage 0.89 and identity 0.69 (both above the cohort median) onto scaffold OY282427.1:12,879,741–12,882,059 (+ strand), immediately downstream of *Acox57D-p* (OY282427.1:12,876,778–12,878,056), reproducing the tandem gene order, orientation, and strand of the *D. melanogaster* 2R locus; the intergenic distance is larger in *D. funebris* (1.7 kb) than in *D. melanogaster* (129 bp), but the tandem synteny of the pair is conserved. As an independent, sequence-based confirmation, we performed a BLASTX search of the *D. funebris Acox57D-d* CDS against the *D. melanogaster Acox* gene family (*Acox57D-d*, *Acox57D-p*, *Acox1*, *Acox3*, *CG4586*, *CG17544*). The top hit was *D. melanogaster Acox57D-d* itself (85.1% amino-acid identity, alignment length 629 aa, E-value = 0), clearly separated from the next-best hit, its tandem paralog *Acox57D-p* (53.4% identity, 624 aa, E-value = 0), and from the more distant family members (49.7%, 42.1%, and 29.0% identity, respectively). Full mapping statistics, the unmapped-gene list, and the BLASTX results table are provided in Supplementary Note S1.

Reads were aligned to the *D. funebris* genome using STAR v2.7.11b [84], and alignment quality was assessed using QualiMap v2.3 and RSeQC v5.0.2. Independently, transcript abundance was quantified using Salmon v1.10.3 [85]. Salmon estimates were imported and aggregated to gene-level count matrices using tximport v1.38.2.

#### 6.3.4. Differential Expression Analysis

Gene-level count matrices were analyzed in R using DESeq2 v1.46.0 [86]. Genes with fewer than 10 total counts across all samples were excluded before analysis. Differential expression was modeled using phenotype (BS versus control) as the design factor. *p* values were adjusted using the Benjamini–Hochberg procedure, and genes with adjusted *p* < 0.05 were considered differentially expressed. Full results are provided in Supplementary Table S2.

### 6.4. Real-Time RT-qPCR

The RT-qPCR protocol was optimized in accordance with the MIQE guidelines [87], following the general laboratory approach described previously [88], with reagent- and gene-specific modifications as detailed below. Total RNA was extracted from pooled samples of 50 heads or 50 bodies per biological replicate, dissected from 10-day-old adult male *Drosophila* funebris. Samples were stored in approximately 10 volumes of ExtractRNA reagent (Evrogen, Russia), and RNA was extracted and subsequently treated with DNase I (Sigma-Aldrich, Darmstadt, Germany) according to the manufacturers’ instructions.

First-strand cDNA was synthesized from approximately 1 microgram of total RNA using the Mint Reverse Transcriptase kit (Evrogen, Russia) and oligo(dT)_15_ primers, according to the manufacturer’s instructions. RT-qPCR was performed with SYBR Green I using a MiniOpticon real-time PCR detection system (Bio-Rad).

The reference genes *Tbp* (TATA-binding protein; FBgn0003687) and *LamC* (Lamin C; FBgn0010397) were selected because they have been reported as stable reference genes in *Drosophila* [89,90,91] and showed expression levels comparable to those of the target gene. Both reference genes showed comparable Cq values across the analyzed samples and were therefore used for dual-reference normalization. The target gene was *Acox57D-d* (acyl-Coenzyme A oxidase at 57D distal), which encodes a peroxisomal fatty acid beta-oxidation enzyme and is expressed at low levels in adult heads and bodies.

The forward and reverse primer sequences used were as follows:

for *Tbp*:
          Tbps, 5′-TATCAAGCGGTCCTGATGGCAGC-3′          Tbpa, 5′-TCATGCTTTGCGGTGTTTGTGGC-3′

for *LamC*:
          LamCs, 5′-ACCGATTCGGGCATCTCCAACAG-3′          LamCa, 5′-AACGTGCGATCCTCGCTCTCATC-3′

for *Acox57D-d*:
          57Dd1: 5′-GCAACCCATAAGACCCGTATTGC-3′          57Dd2: 5′-TGTTGAGCACAGTCTGCACTAGGA-3′

Relative *Acox57D-d* mRNA abundance was calculated in CFX Manager v3.1 software (Bio-Rad) using an efficiency-corrected comparative approach. Experimentally determined amplification efficiencies were 90% for *Acox57D-d*, 95% for *LamC*, and 97.5% for *Tbp*. For each biological replicate, target-gene expression was normalized to the geometric mean of the two reference targets, *Tbp* and *LamC*, following the multi-reference normalization principle described by Vandesompele et al. [92,93].

Five independent RNA extractions were analyzed for each genotype-by-tissue group, with three technical RT-qPCR replicates per RNA extraction. Technical replicates were averaged before the calculation of normalized relative expression, and each biological replicate contributed one value to the statistical analysis. Normality was assessed separately in the four genotype-by-tissue groups (control heads, BS heads, control bodies, and BS bodies) using the Shapiro–Wilk test. Because no group showed a statistically significant departure from normality, prespecified control-versus-BS comparisons were performed separately in heads and bodies using two-sided independent-samples *t*-tests. In addition, a two-way analysis of variance (ANOVA) with genotype, tissue, and the genotype × tissue interaction as fixed effects was performed using all biological replicates to test whether the genotype effect differed between tissues. Statistical significance was set at *p* < 0.05.

### 6.5. Lipidomic Analysis

Lipidomic profiling was performed on the heads of *Drosophila funebris* ten-day-old males. On each of three independent collection days, two separate pools of 15 intact heads were collected per group (BS and control), yielding two independently collected biological samples per day and six independent biological samples per group in total (rep_1a and rep_1b from day 1, rep_2a and rep_2b from day 2, rep_3a and rep_3b from day 3). Each of these six samples was extracted and processed for lipid analysis independently, from a distinct pool of 15 flies; the “a” and “b” labels denote two independently collected and independently extracted biological samples obtained on the same day, not technical replicates or aliquots of a single extraction. This design allowed the six biological replicates per group (*n* = 6) used in downstream statistical analysis to capture both within-day and between-day biological variability.

The samples were stored at −80 °C until processing. Prior to extraction, the samples were thawed on ice and transferred to bead-beating tubes containing four 2.38 mm steel balls. Lipids were extracted using 1000 µL of Folch solution (chloroform:methanol, 2:1, *v*/*v*) [94] supplemented with Avanti SPLASH LipidoMix mass spectrometry internal standard. Homogenization was performed using a bead-bluster homogenizer (3 cycles, 7000 rpm, 20 s per cycle, with 1 min cooling intervals). Homogenates were centrifuged at 13,000 rpm for 10 min at 10 °C, and 700 µL of the lower organic phase (supernatant) was collected and evaporated at 20 °C under a gentle nitrogen stream. The dried residue was reconstituted in 70 µL of mobile phase B, centrifuged again at 13,000 rpm for 10 min at 10 °C, and 50 µL aliquots were transferred to glass vial inserts for LC-MS analysis.

Chromatographic separation was performed on an Agilent Infinity II 1290 UHPLC system using an Agilent Eclipse Plus C18 RRHD column (2.1 × 100 mm, 1.8 µm) operated in reverse-phase mode at 60 °C. Mobile phase A consisted of water:acetonitrile (4:6, *v*/*v*) with 10 mM ammonium acetate and 0.2 mM ammonium fluoride; mobile phase B consisted of acetonitrile:isopropanol (1:9, *v*/*v*) with the same additives. The gradient elution was as follows: 0 min, 15% B; 2 min, 30% B; 2.5 min, 48% B; 8.5 min, 72% B; 11.5 min, 99% B; 13 min, 99% B; 13.1 min, 15% B; 16 min, 15% B. The flow rate was 0.45 mL/min and the injection volume was 2 µL.

Mass spectrometric detection was performed on an Agilent 6546 quadrupole time-of-flight (Q-TOF) instrument operating in information-dependent acquisition (IDA) mode, scanning from *m/z* 350 to 2000 in both positive and negative ionization modes. ESI source parameters were as follows: capillary voltage (VCap) 3500 V, fragmentor 125 V, nebulizer pressure 35 psi, drying gas temperature 200 °C, drying gas flow 8 L/min, sheath gas temperature 350 °C, sheath gas flow 11 L/min, octopole RF 750 V.

Raw data were acquired using MassHunter Acquisition Software v12.1 (Agilent Technologies, Santa Clara, CA, USA). Lipid identification and structural annotation were performed in MS-DIAL v4.9 [95] using in silico-generated MS/MS spectral libraries based on the LipidBlast database [96] supplemented with proprietary MS-DIAL in silico libraries. Quantitative peak integration and verification were carried out in Skyline v23 [97].

Blank samples were included at the beginning and end of the analytical sequence to monitor contamination during sample preparation and potential carry-over or cross-contamination during LC–MS analysis. Pooled quality-control samples were not included. Before analysis of the experimental series, LC–MS performance was evaluated using a standard control sample as part of the routine daily system check. The retention times and signal intensities of isotope-labeled internal standards were monitored throughout the analytical sequence. A strict randomization of injection order was not performed. All lipid extracts were prepared and analyzed within a single day, thereby minimizing potential batch and day effects.

Raw LC–MS data were processed in MS-DIAL v4.9. Features with peak heights below 1000 arbitrary units were excluded. Features whose intensity in biological samples was less than 5-fold higher than in blank samples were excluded as background or potential contamination. MS1 and MS/MS mass tolerances were both set to 0.025 Da. The retention-time tolerance used for chromatographic alignment across samples was 0.1 min. Lipid annotation was performed by automated matching of experimental MS/MS spectra against the LipidBlast-based spectral libraries integrated in MS-DIAL. The matching algorithm considered precursor mass accuracy, diagnostic fragment mass accuracy, relative fragment-intensity similarity, and isotope-pattern agreement. Annotations with an MS-DIAL total score of at least 80% were retained. Retention-time matching against authentic reference standards was not used; accordingly, lipid identifications are reported as putative annotations based on accurate mass and MS/MS library matching.

Peak intensities were normalized to account for analytical variation. For each sample, the intensity of each detected lipid feature was divided by the summed intensities of isotope-labeled SPLASH LipidoMix internal standards detected in the corresponding ionization mode. This normalization was performed separately for positive and negative ionization datasets. The resulting values are referred to as normalized peak areas. The SPLASH LipidoMix standards were used for global within-mode signal normalization and were not used for absolute quantification or individual lipid-class-specific calibration.

Statistical comparisons of normalized peak areas between BS and control samples were performed for all detected lipid species using two-sided Mann–Whitney U tests. The resulting *p* values were adjusted for multiple comparisons using the Benjamini–Hochberg procedure across all 294 detected lipid species. Lipid species with BH-adjusted *p* values < 0.05 were considered statistically significant.

## Data Availability

The RNA-seq data generated in this study have been deposited in NCBI’s Gene Expression Omnibus (GEO) database and are accessible under accession number GSE343916 (https://www.ncbi.nlm.nih.gov/geo/query/acc.cgi?acc=GSE343916, accessed on 2 September 2026). All lipidomic data, differential expression results, and GO enrichment analyses supporting the findings of this study are provided within the article and its Supplementary Materials (Supplementary Tables S2–S4). Any additional data are available from the corresponding author upon reasonable request.

## Supplementary Materials

The following supporting information can be downloaded at: https://www.mdpi.com/article/10.3390/ijms27198845/s1.

## References

[1] Ganetzky B., Wu C.-F. (1982). Indirect suppression involving behavioral mutants with altered nerve excitability in *drosophila melanogaster*. Genetics.

[2] Pavlidis P., Tanouye M. (1995). Seizures and failures in the giant fiber pathway of Drosophila bang- sensitive paralytic mutants. J. Neurosci..

[3] Fischer F.P., Karge R.A., Weber Y.G., Koch H., Wolking S., Voigt A. (2023). Drosophila melanogaster as a versatile model organism to study genetic epilepsies: An overview. Front. Mol. Neurosci..

[4] Howlett I.C., Rusan Z.M., Parker L., Tanouye M.A. (2013). *Drosophila* as a Model for Intractable Epilepsy: *Gilgamesh* Suppresses Seizures in *para^bss1^* Heterozygote Flies. G3 Genes|Genomes|Genetics.

[5] Saras A., Tanouye M.A. (2016). Mutations of the Calcium Channel Gene cacophony Suppress Seizures in Drosophila. PLoS Genet..

[6] Dibbens L.M., Heron S.E., Mulley J.C. (2007). A polygenic heterogeneity model for common epilepsies with complex genetics. Genes Brain Behav..

[7] Kuebler D., Tanouye M.A. (2000). Modifications of Seizure Susceptibility in *Drosophila*. J. Neurophysiol..

[8] Amichot M., Castella C., Bergé J.-B., Pauron D. (1993). Transcription analysis of the para gene by in situ hybridization and immunological characterization of its expression product in wild-type and mutant strains of Drosophila. Insect Biochem. Mol. Biol..

[9] Parker L., Padilla M., Du Y., Dong K., Tanouye M.A. (2011). Drosophila as a Model for Epilepsy: *bss* Is a Gain-of-Function Mutation in the Para Sodium Channel Gene That Leads to Seizures. Genetics.

[10] Pavlidis P., Ramaswami M., Tanouye M.A. (1994). The Drosophila easily shocked gene: A mutation in a phospholipid synthetic pathway causes seizure, neuronal failure, and paralysis. Cell.

[11] Royden C. (1987). The *tko* locus, site of a behavioral mutation in *D. melanogaster*, codes for a protein homologous to prokaryotic ribosomal protein S12. Cell.

[12] Toivonen J.M., O’DEll K.M.C., Petit N., Irvine S.C., Knight G.K., Lehtonen M., Longmuir M., Luoto K., Touraille S., Wang Z. (2001). *technical knockout*, a Drosophila Model of Mitochondrial Deafness. Genetics.

[13] Zhang Y.Q., Roote J., Brogna S., Davis A.W., Barbash D.A., Nash D., Ashburner M. (1999). stress sensitive B Encodes an Adenine Nucleotide Translocase in Drosophila melanogaster. Genetics.

[14] Terhzaz S., Cabrero P., Chintapalli V.R., Davies S.-A., Dow J.A.T. (2010). Mislocalization of mitochondria and compromised renal function and oxidative stress resistance in *Drosophila SesB* mutants. Physiol. Genom..

[15] Fergestad T., Bostwick B., Ganetzky B. (2006). Metabolic Disruption in Drosophila Bang-Sensitive Seizure Mutants. Genetics.

[16] Fergestad T., Olson L., Patel K.P., Miller R., Palladino M.J., Ganetzky B. (2008). Neuropathology in Drosophila Mutants with Increased Seizure Susceptibility. Genetics.

[17] Zhang H., Tan J., Reynolds E., Kuebler D., Faulhaber S., Tanouye M. (2002). The Drosophila *slamdance* Gene: A Mutation in an Aminopeptidase Can Cause Seizure, Paralysis and Neuronal Failure. Genetics.

[18] Horne M., Krebushevski K., Wells A., Tunio N., Jarvis C., Francisco G., Geiss J., Recknagel A., Deitcher D.L. (2017). *julius seizure*, a *Drosophila* Mutant, Defines a Neuronal Population Underlying Epileptogenesis. Genetics.

[19] Schubiger M., Feng Y., Fambrough D.M., Palka J. (1994). A mutation of the drosophila sodium pump α subunit gene results in bang-sensitive paralysis. Neuron.

[20] Hope K.A., LeDoux M.S., Reiter L.T. (2017). Glial overexpression of Dube3a causes seizures and synaptic impairments in Drosophila concomitant with down regulation of the Na+/K+ pump ATPα. Neurobiol. Dis..

[21] Terry B.K., Mahoney A., Lee B.I., Secombe J. (2026). Individuals with reported and novel *KDM5C* variants present with seizures, a feature recapitulated in a *Drosophila* model. Hum. Mol. Genet..

[22] Shah N., Kasture A.S., Kovalchuk V., Bjeletic L., Hummel T., Sitte H.H., Sucic S. (2025). Pathogenic variants at the N-terminal arginine residue 44 disrupt human GABA transporter 1 function: Insights from Drosophila epilepsy models. Front. Pharmacol..

[23] Karge R.A., Fischer F.P., Schüth H., Wechner A., Peter S., Kilo L.A., Dichter M., Voigt A., Tavosanis G., van Loo K.M.J. (2025). Modeling AP2M1 developmental and epileptic encephalopathy in *Drosophila*. Dis. Model. Mech..

[24] Parker L., Howlett I.C., Rusan Z.M., Tanouye M.A. (2011). Seizure and Epilepsy: Studies of Seizure Disorders in Drosophila.

[25] Ricos M.G., Cole B.A., Hussain R., Rychkov G.Y., Shaukat Z., Pilati N., Muench S.P., Simmons K.J., Dibbens L.M., Lippiat J.D. (2025). Identification of New KCNT1-Epilepsy Drugs by In Silico, Cell, and *Drosophila* Modeling. Ann. Neurol..

[26] Töfflinger E.V., Makridis K.L., Heim M., Owald D., Kaindl A.M. (2025). *Drosophila melanogaster* as a rapid in vivo assay system for preclinical anti-seizure medication testing. Epilepsia Open.

[27] Song J., Tanouye M. (2008). From bench to drug: Human seizure modeling using Drosophila. Prog. Neurobiol..

[28] Humphreys R.K., Ruxton G.D. (2018). A review of thanatosis (death feigning) as an anti-predator behaviour. Behav. Ecol. Sociobiol..

[29] Ailion A., Helmstaedter C., Vezzani A., Koepp M.J. (2025). The upside of epilepsy: Theories of an evolutionary paradox. Epilepsia Open.

[30] Grimaldi D.A. (2022). The Drosophila funebris Species Group in North America (Diptera: Drosophilidae). Am. Mus. Novit..

[31] Timoféeff-Ressovsky N.W. (1927). Studies on the Phenotypic Manifestation of Hereditary Factors. I. on the Phenotypic Manifestation of the Genovariation Radius Incompletus in *Drosophila funebris*. Genetics.

[32] Obbard D.J. (2023). Wellcome Sanger Institute Tree of Life Programme; Wellcome Sanger Institute Scientific Operations: DNA Pipelines collective; Tree of Life Core Informatics collective; Darwin Tree of Life Consortium. The genome sequence of a drosophilid fruit fly, *Drosophila funebris* (Fabricius, 1789). Wellcome Open Res..

[33] Shumate A., Salzberg S.L. (2021). Liftoff: Accurate mapping of gene annotations. Bioinformatics.

[34] Ratnasingham S., Wei C., Chan D., Agda J., Agda J., Ballesteros-Mejia L., Boutou H.A., El Bastami Z.M., Ma E., Manjunath R. (2024). BOLD v4: A Centralized Bioinformatics Platform for DNA-Based Biodiversity Data.

[35] Ratnasingham S., Hebert P.D.N. (2007). BOLD: The Barcode of Life Data System (http://www.barcodinglife.org). Mol. Ecol. Notes.

[36] Hu Y., Comjean A., Attrill H., Antonazzo G., Thurmond J., Chen W., Li F., Chao T., Mohr S.E., Brown N.H. (2023). PANGEA: A new gene set enrichment tool for *Drosophila* and common research organisms. Nucleic Acids Res..

[37] Hu Y., Comjean A., Rodiger J., Chen W., Kim A.-R., Qadiri M., Gao C., Zirin J., Mohr S.E., Perrimon N. (2024). FlyRNAi.org 2025 update—Expanded resources for new technologies and species. Nucleic Acids Res..

[38] Okamura T., Shimizu H., Nagao T., Ueda R., Ishii S. (2007). ATF-2 Regulates Fat Metabolism in *Drosophila*. Mol. Biol. Cell.

[39] Eveland M., Brokamp G.A., Lue C.-H., Harbison S.T., Leips J., De Luca M. (2016). Knockdown expression of Syndecan in the fat body impacts nutrient metabolism and the organismal response to environmental stresses in Drosophila melanogaster. Biochem. Biophys. Res. Commun..

[40] Kranendijk M., Struys E.A., Salomons G.S., Van der Knaap M.S., Jakobs C. (2012). Progress in understanding 2-hydroxyglutaric acidurias. J. Inherit. Metab. Dis..

[41] Zhang Z., Zhang H., Zhang P., Li R., Zhou J., Li J., Hu D., Huang R., Tang F., Liu J. (2024). *D2HGDH* Deficiency Regulates Seizures through GSH/Prdx6/ROS-Mediated Excitatory Synaptic Activity. Adv. Sci..

[42] Chung H., Bogwitz M.R., McCart C., Andrianopoulos A., Ffrench-Constant R.H., Batterham P., Daborn P.J. (2007). Cis-Regulatory Elements in the *Accord* Retrotransposon Result in Tissue-Specific Expression of the *Drosophila melanogaster* Insecticide Resistance Gene *Cyp6g1*. Genetics.

[43] Heier C., Kühnlein R.P. (2018). Triacylglycerol Metabolism in *Drosophila melanogaster*. Genetics.

[44] Schulz J.G., Laranjeira A., Van Huffel L., Gärtner A., Vilain S., Bastianen J., Van Veldhoven P.P., Dotti C.G. (2015). Glial β-Oxidation regulates Drosophila Energy Metabolism. Sci. Rep..

[45] Islimye E., Girard V., Gould A.P. (2022). Functions of Stress-Induced Lipid Droplets in the Nervous System. Front. Cell Dev. Biol..

[46] Ren M., Xu Y., Phoon C.K., Erdjument-Bromage H., Neubert T.A., Schlame M. (2023). Cardiolipin prolongs the lifetimes of respiratory proteins in Drosophila flight muscle. J. Biol. Chem..

[47] Zhao H., Wang T. (2020). PE homeostasis rebalanced through mitochondria-ER lipid exchange prevents retinal degeneration in Drosophila. PLoS Genet..

[48] Waterham H.R., Ferdinandusse S., Wanders R.J. (2016). Human disorders of peroxisome metabolism and biogenesis. Biochim. Biophys. Acta (BBA) Mol. Cell Res..

[49] Okumoto K., El Shermely M., Natsui M., Kosako H., Natsuyama R., Marutani T., Fujiki Y. (2020). The peroxisome counteracts oxidative stresses by suppressing catalase import via Pex14 phosphorylation. eLife.

[50] Sanders R., Ofman R., Dacremont G., Wanders R.J.A., Kemp S. (2008). Characterization of the human ω-oxidation pathway for ω-hydroxy-very-long-chain fatty acids. FASEB J..

[51] Miura Y. (2013). The biological significance of ω-oxidation of fatty acids. Proc. Jpn. Acad. Ser. B.

[52] Sanders R.-J., Ofman R., Duran M., Kemp S., Wanders R.J. (2006). ω-Oxidation of Very Long-chain Fatty Acids in Human Liver Microsomes. J. Biol. Chem..

[53] Sellin J., Wingen C., Gosejacob D., Senyilmaz D., Hänschke L., Büttner S., Meyer K., Bano D., Nicotera P., Teleman A.A. (2018). Dietary rescue of lipotoxicity-induced mitochondrial damage in Peroxin19 mutants. PLoS Biol..

[54] Rosca M.G., Vazquez E.J., Kerner J., Parland W., Chandler M.P., Stanley W., Sabbah H.N., Hoppel C.L. (2008). Cardiac mitochondria in heart failure: Decrease in respirasomes and oxidative phosphorylation. Cardiovasc. Res..

[55] Carrasco S., Mérida I. (2007). Diacylglycerol, when simplicity becomes complex. Trends Biochem. Sci..

[56] Eichmann T.O., Lass A. (2015). DAG tales: The multiple faces of diacylglycerol—Stereochemistry, metabolism, and signaling. Cell. Mol. Life Sci..

[57] Lingwood D., Simons K. (2010). Lipid Rafts As a Membrane-Organizing Principle. Science.

[58] Goñi F.M., Alonso A. (1999). Structure and functional properties of diacylglycerols in membranes ^1^. Prog. Lipid Res..

[59] Wassall S.R., Stillwell W. (2009). Polyunsaturated fatty acid–cholesterol interactions: Domain formation in membranes. Biochim. Biophys. Acta (BBA)—Biomembr..

[60] Chwastek G., Surma M.A., Rizk S., Grosser D., Lavrynenko O., Rucińska M., Jambor H., Sáenz J. (2020). Principles of Membrane Adaptation Revealed through Environmentally Induced Bacterial Lipidome Remodeling. Cell Rep..

[61] Wanders R.J., Waterham H.R. (2006). Biochemistry of Mammalian Peroxisomes Revisited. Annu. Rev. Biochem..

[62] Listenberger L.L., Han X., Lewis S.E., Cases S., Farese R.V., Ory D.S., Schaffer J.E. (2003). Triglyceride accumulation protects against fatty acid-induced lipotoxicity. Proc. Natl. Acad. Sci. USA.

[63] Di Cara F., Savary S. (2026). Peroxisomes and the nervous system: Progress and challenges in neuroinflammation and cell-specific functions. Front. Mol. Neurosci..

[64] Barry D.S., O’kEeffe G.W. (2013). Peroxisomes: The neuropathological consequences of peroxisomal dysfunction in the developing brain. Int. J. Biochem. Cell Biol..

[65] Leader D.P., Krause S.A., Pandit A., Davies S.A., Dow J.A.T. (2017). FlyAtlas 2: A new version of the Drosophila melanogaster expression atlas with RNA-Seq, miRNA-Seq and sex-specific data. Nucleic Acids Res..

[66] Jang H.J., Park J.H., Lee G.S., Lee S.B., Moon J.H., Choi J.S., Lee T.G., Yoon S. (2019). Comparison of Lipid Profiles in Head and Brain Samples of Drosophila Melanogaster Using Electrospray Ionization Mass Spectrometry (ESI-MS). Mass Spectrom. Lett..

[67] Mayar S., Borbuliak M., Zoumpoulakis A., Bouceba T., Labonté M.M., Ahrari A., Sinniah N., Memarpoor-Yazdi M., Vénien-Bryan C., Tieleman D.P. (2024). Endocannabinoid regulation of inward rectifier potassium (Kir) channels. Front. Pharmacol..

[68] Wanders R.J.A., Waterham H.R., Ferdinandusse S. (2016). Metabolic Interplay between Peroxisomes and Other Subcellular Organelles Including Mitochondria and the Endoplasmic Reticulum. Front. Cell Dev. Biol..

[69] Yin H., Zhu M. (2012). Free radical oxidation of cardiolipin: Chemical mechanisms, detection and implication in apoptosis, mitochondrial dysfunction and human diseases. Free Radic. Res..

[70] Sen T., Sen N., Tripathi G., Chatterjee U., Chakrabarti S. (2006). Lipid peroxidation associated cardiolipin loss and membrane depolarization in rat brain mitochondria. Neurochem. Int..

[71] Paradies G., Paradies V., Ruggiero F.M., Petrosillo G. (2014). Oxidative stress, cardiolipin and mitochondrial dysfunction in nonalcoholic fatty liver disease. World J. Gastroenterol..

[72] Joshi A.S., Thompson M.N., Fei N., Hüttemann M., Greenberg M.L. (2012). Cardiolipin and Mitochondrial Phosphatidylethanolamine Have Overlapping Functions in Mitochondrial Fusion in Saccharomyces cerevisiae. J. Biol. Chem..

[73] Pope S., Land J.M., Heales S.J. (2008). Oxidative stress and mitochondrial dysfunction in neurodegeneration; cardiolipin a critical target?. Biochim. Biophys. Acta (BBA)—Bioenerg..

[74] Sato A., Ohhara Y., Miura S., Yamakawa-Kobayashi K. (2020). The presence of odd-chain fatty acids in *Drosophila* phospholipids. Biosci. Biotechnol. Biochem..

[75] Shin S.C., Kim S.-H., You H., Kim B., Kim A.C., Lee K.-A., Yoon J.-H., Ryu J.-H., Lee W.-J. (2011). Drosophila Microbiome Modulates Host Developmental and Metabolic Homeostasis via Insulin Signaling. Science.

[76] Kamareddine L., Robins W.P., Berkey C.D., Mekalanos J.J., Watnick P.I. (2018). The Drosophila Immune Deficiency Pathway Modulates Enteroendocrine Function and Host Metabolism. Cell Metab..

[77] Song W., Veenstra J.A., Perrimon N. (2014). Control of Lipid Metabolism by Tachykinin in Drosophila. Cell Rep..

[78] Chung H.-L., Wangler M.F., Marcogliese P.C., Jo J., Ravenscroft T.A., Zuo Z., Duraine L., Sadeghzadeh S., Li-Kroeger D., Schmidt R.E. (2020). Loss- or Gain-of-Function Mutations in ACOX1 Cause Axonal Loss via Different Mechanisms. Neuron.

[79] Takahashi Y., Suzuki Y., Kumazaki K., Tanabe Y., Akaboshi S., Miura K., Shimozawa N., Kondo N., Nishiguchi T., Terada K. (1997). Epilepsy in Peroxisomal Diseases. Epilepsia.

[80] Schurch N.J., Schofield P., Gierliński M., Cole C., Sherstnev A., Singh V., Wrobel N., Gharbi K., Simpson G.G., Owen-Hughes T. (2016). How many biological replicates are needed in an RNA-seq experiment and which differential expression tool should you use?. RNA.

[81] Lansdon P., Kasuya J., Kitamoto T. (2024). Commensal bacteria exacerbate seizure-like phenotypes in *Drosophila* voltage-gated sodium channel mutants. Genes Brain Behav..

[82] Hebert P.D.N., Cywinska A., Ball S.L., Dewaard J.R. (2003). Biological identifications through DNA barcodes. Proc. R. Soc. Lond. Ser. B Biol. Sci..

[83] Sanger F., Nicklen S., Coulson A.R. (1977). DNA sequencing with chain-terminating inhibitors. Proc. Natl. Acad. Sci. USA.

[84] Dobin A., Davis C.A., Schlesinger F., Drenkow J., Zaleski C., Jha S., Batut P., Chaisson M., Gingeras T.R. (2012). STAR: Ultrafast universal RNA-seq aligner. Bioinformatics.

[85] Patro R., Duggal G., Love M.I., Irizarry R.A., Kingsford C. (2017). Salmon provides fast and bias-aware quantification of transcript expression. Nat. Methods.

[86] Love M.I., Huber W., Anders S. (2014). Moderated estimation of fold change and dispersion for RNA-seq data with DESeq2. Genome Biol..

[87] Bustin S.A., Benes V., Garson J.A., Hellemans J., Huggett J., Kubista M., Mueller R., Nolan T., Pfaffl M.W., Shipley G.L. (2009). The MIQE Guidelines: Minimum Information for Publication of Quantitative Real-Time PCR Experiments. Clin. Chem..

[88] Symonenko A.V., Roshina N.V., Krementsova A.V., Pasyukova E.G. (2018). Reduced Neuronal Transcription of Escargot, the Drosophila Gene Encoding a Snail-Type Transcription Factor, Promotes Longevity. Front. Genet..

[89] De Groef S., Lopes M.R., Winant M., Rosschaert E., Wilms T., Bolckmans L., Calevro F., Callaerts P. (2024). Reference genes to study the sex-biased expression of genes regulating Drosophila metabolism. Sci. Rep..

[90] Matta B.P., Bitner-Mathé B.C., Alves-Ferreira M. (2011). Getting real with real-time qPCR: A case study of reference gene selection for morphological variation in Drosophila melanogaster wings. Dev. Genes Evol..

[91] Zhai Y., Lin Q., Zhou X., Zhang X., Liu T., Yu Y. (2014). Identification and Validation of Reference Genes for Quantitative Real-Time PCR in Drosophila suzukii (Diptera: Drosophilidae). PLoS ONE.

[92] Vandesompele J., De Preter K., Pattyn F., Poppe B., Van Roy N., De Paepe A., Speleman F. (2002). Accurate normalization of real-time quantitative RT-PCR data by geometric averaging of multiple internal control genes. Genome Biol..

[93] Livak K.J., Schmittgen T.D. (2001). Analysis of relative gene expression data using real-time quantitative PCR and the 2^−ΔΔCT^ Method. Methods.

[94] Folch J., Lees M., Stanley G.H.S. (1957). A simple method for the isolation and purification of total lipides from animal tissues. J. Biol. Chem..

[95] Tsugawa H., Cajka T., Kind T., Ma Y., Higgins B., Ikeda K., Kanazawa M., VanderGheynst J., Fiehn O., Arita M. (2015). MS-DIAL: Data-independent MS/MS deconvolution for comprehensive metabolome analysis. Nat. Methods.

[96] Kind T., Liu K.-H., Lee D.Y., DeFelice B., Meissen J.K., Fiehn O. (2013). LipidBlast in silico tandem mass spectrometry database for lipid identification. Nat. Methods.

[97] MacLean B., Tomazela D.M., Shulman N., Chambers M., Finney G.L., Frewen B., Kern R., Tabb D.L., Liebler D.C., MacCoss M.J. (2010). Skyline: An open source document editor for creating and analyzing targeted proteomics experiments. Bioinformatics.

