# Supplementary material for: The First Report of a Bang-Sensitive Strain in Drosophila funebris: Molecular-Genetic and Lipidomic Characterization
Source: Int J Mol Sci. 2026 Oct 3;27(19):8845. doi: 10.3390/ijms27198845 (PMC13665471; doi:10.3390/ijms27198845)
Supplement: Supplementary file 1 [file ijms-27-08845-s001.zip › Supplementary Note S1.pdf]

Supplementary Note S1. Liftoff annotation transfer: mapping parameters, summary statistics, and orthology confirmation for *Acox57D-d*.

1. Mapping command and parameters

The *D. melanogaster* reference annotation (GCF_000001215.4, Release 6 plus ISO1 MT) was transferred to the D. funebris genome assembly (GCA_958295475.1, idDroFune2.1) using Liftoff v1.6.3 [Shumate & Salzberg, 2021], run as follows:

```
liftoff -g GCF_000001215.4/genomic.gff \
        -o GCA_958295475.1/genomic_liftoff.gff \
        -u GCA_958295475.1/genomic_liftoff_unmapped.gff \
        -polish -p 64 \
        GCA_958295475.1_idDroFune2.1_genomic.fna \
        GCF_000001215.4_Release_6_plus_ISO1_MT_genomic.fna
```

Liftoff was run with its default mapping thresholds:
- Minimum sequence identity: 0.5
- Minimum alignment coverage: 0.5
- -polish option enabled (re-aligns and refines CDS boundaries after initial mapping)
- 64 parallel processes (-p 64)

Under Liftoff's default behavior, partial mappings are retained rather than discarded at the mapping stage; coverage and identity values are reported per feature so that they can be inspected and, if desired, filtered downstream. No additional post hoc filtering beyond Liftoff's own default thresholds was applied in this study, so that all transferred protein-coding genes discussed in the manuscript, including *Acox57D-d*, are reported with their native Liftoff quality metrics.

2. Genome-wide summary statistics

Metric	Value
Protein-coding genes in *D. melanogaster* reference (GCF_000001215.4)	13,981
Protein-coding genes successfully transferred to *D. funebris*	4,643 (33.2%)
Protein-coding genes unmapped / below threshold	9,338 (66.8%)
Median alignment coverage (transferred genes)	0.81
Median sequence identity (transferred genes)	0.60
Genes flagged partial_mapping by Liftoff	20.4%
Genes flagged low_identity by Liftoff	24.5%

The full list of unmapped features is provided in the accompanying genomic_liftoff_unmapped.gff file (generated directly by Liftoff via the -u flag) and is available from the corresponding author upon request together with the complete annotation transfer output.

Given this mapping quality profile, we note explicitly that individual gene-level annotations transferred by Liftoff, including but not limited to Acox57D-d, should be interpreted with appropriate caution, particularly for genes with coverage or identity near the lower end of this distribution.

3. Mapping quality and orthology confirmation for Acox57D-d

3.1 Liftoff mapping statistics

Acox57D-d mapped at coverage 0.89 and identity 0.69, both above the cohort median (0.81 and 0.60, respectively), onto scaffold OY282427.1:12,879,741-12,882,059 (+ strand) of the D. funebris assembly.

3.2 Synteny confirmation

The *D. funebris* locus reproduces the gene order, orientation, and strand of the corresponding D. melanogaster 2R locus:

	D. melanogaster (2R)	*D. funebris* (OY282427.1)
Acox57D-p position	upstream	12,876,778-12,878,056
Acox57D-d position	downstream	12,879,741-12,882,059
Strand	+	+
Intergenic distance	129 bp	1.7 kb

The tandem arrangement and relative order of the paralog pair (*Acox57D-p* immediately upstream of *Acox57D-d*) are conserved between species, despite a substantially larger intergenic distance in *D. funebris*.

3.3 Sequence-based confirmation (BLASTX)

The *D. funebris Acox57D-d* coding sequence (CDS) was searched by BLASTX against the *D. melanogaster Acox* gene family (*Acox57D-d*, *Acox57D-p*, *Acox1*, *Acox3*, *CG4586*, *CG17544*).

Query	Subject (*D. melanogaster*)	% identity	Alignment length (aa)	E-value
D. funebris Acox57D-d CDS	*Acox57D-d* (UniProt Q9W2G8)	85.06	629	0
D. funebris Acox57D-d CDS	*Acox57D-p* (UniProt Q9W2G9), tandem paralog	53.37	624	0
D. funebris Acox57D-d CDS	*Acox1* (UniProt Q7KML2)	49.67	614	0
D. funebris Acox57D-d CDS	*Acox* family member (UniProt Q9W3U0)	42.11	646	1.85e-159
D. funebris Acox57D-d CDS	*Acox* family member (UniProt Q9VIX4)	29.03	565	7.29e-61

The top BLASTX hit is unambiguously *D. melanogaster Acox57D-d* (85.1% amino-acid identity), with a clear identity gap (>31 percentage points) separating

it from the next-best hit, its tandem paralog *Acox57D-p* (53.4% identity). This sequence-level result is fully consistent with the annotation transferred by Liftoff and with the conserved synteny described in Section 3.2.

4. Conclusion

The above-median Liftoff mapping quality for *Acox57D-d* (coverage 0.89, identity 0.69), the conserved tandem synteny with *Acox57D-p* across a substantially expanded intergenic distance, and the unambiguous BLASTX top hit to *D. melanogaster Acox57D-d* (85.1% identity, well separated from the 53.4% identity of the paralog) provide three independent, convergent lines of evidence supporting the orthology assignment of *Acox57D-d* in *D. funebris* used throughout this study.
