# Supplementary material for: The First Report of a Bang-Sensitive Strain in Drosophila funebris: Molecular-Genetic and Lipidomic Characterization
Source: Int J Mol Sci. 2026 Oct 3;27(19):8845. doi: 10.3390/ijms27198845 (PMC13665471; doi:10.3390/ijms27198845)
Supplement: Supplementary file 1 [file ijms-27-08845-s001.zip › Suppl_Table S1.pdf]

Supplementary Table S1. Genetically characterized bang-sensitive (BS) loci in *Drosophila melanogaster*

Gene symbol	Allele	Full name	Chr.	Cytol. position	Protein product	Mutation type	AA change	Inheritance	BS phenotype	FlyBase ID	Ref.
para (bss)	*$para^{bss1}$*	*paralytic / bangsenseless*	X	14C8-14E1	Voltage-gated Na^+ channel (a-subunit)	GOF	L1699F	semi-dominant	Full (severe)	FBgn0285944	[1, 8, 9]
**eas**	*eas^1*	*easily shocked*	X	14B7	Ethanolamine kinase	LOF	Frameshift	recessive	Full	FBgn0000536	[10]
tko	*tko^{ts}*	*technical knockout*	X	3A3	Mitochondrial ribosomal protein S12	LOF	Missense	recessive	Full	FBgn0003714	[11, 12]
sesB	*$sesB^1$*	*stress-sensitive B*	X	9E10-9F1	Adenine nucleotide translocase (ANT)	LOF	Missense	recessive	Full	FBgn0003360	[13, 14]
kdn	*kdn^1*	*knockdown*	X	5F2-5F4	Citrate synthase	LOF	Missense	recessive	Full (incomplete penetrance)	FBgn0261955	[15, 16]
jus (sda)	*jus^1*	*julius seizure / slamdance*	3R	98F13-99A1	Transmembrane protein (Na^+/K^+-ATPase complex stabilizer)	LOF	Frameshift	recessive	Full	FBgn0039647	[1, 17, 18]

ATPα	P-element enhancer-trap insertion (93B1-2)	*Na pump α subunit*	3L	93A4	Na^+/K^+-ATPase α-subunit	LOF	—	recessive	Partial (Modifier)	FBgn0002921	[19, 20]
bas	*bas^{1}*	*bang sensitive*	—	—	Not identified	Unknown	—	recessive	Full	FBgn0000160	[1]

Abbreviations: Chr., chromosome; Cytol., cytological; GOF, gain-of-function; LOF, loss-of-function; AA, amino acid; BS, bang-sensitive; Ref., reference.

Notes:

- For $para^{bss1}$, the cited literature establishes its molecular identity as a hypermorphic (gain-of-function) allele of the voltage-gated sodium channel gene *paralytic (para)*.
- The *slamdance (sda)* locus was historically misidentified as encoding aminopeptidase N; subsequent refined genetic analysis demonstrated that the causative mutation resides in the neighboring gene *julius seizure (jus*, formerly *CG14509*), which encodes a transmembrane protein interacting directly with the Na^+/K^+-ATPase complex. Consequently, the former *sda* and current *jus* entries are unified into a single physical locus sharing FlyBase ID FBgn0039647.
- *ATPα* heterozygotes display a mild BS phenotype and are primarily classified as systemic modifiers of membrane excitability rather than classical primary structural BS loci.
- The *kdn* locus displays incomplete penetrance and highly variable behavioral expressivity.

Functional Classification of *Drosophila melanogaster* Bang-Sensitive (BS) Mutants

Group 1: Mitochondrial Bioenergetic Crisis (ATP Starvation)

Mutations within this category directly impair mitochondrial homeostasis, depriving cells of metabolic energy. In the absence of sufficient ATP pools, active ion pumps fail to operate, inevitably triggering metabolic synaptic arrest and paralysis.

- ***tko*** **(*technical knockout*):** A mutation in the mitochondrial ribosomal protein S12. It selectively disrupts the translation of core electron transport chain (ETC) subunits, leading to a profound collapse in cellular ATP synthesis.
- ***sesB*** **(*stress-sensitive B*):** A mutation encoding the mitochondrial adenine nucleotide translocase (ANT). It blocks the translocation of newly synthesized ATP from the mitochondrial matrix into the synaptosomal cytosol.

- ***kdn*** **(*knockdown*):** A mutation in citrate synthase, a rate-limiting enzyme of the tricarboxylic acid (TCA) cycle. It halts the downstream supply of reducing equivalents to the respiratory chain, precipitating acute energetic starvation.
- ***eas*** **(*easily shocked*):** Positioned at the metabolic intersection. It disrupts the biosynthesis of phosphatidylethanolamine (PE), which compromises mitochondrial inner membrane integrity and secondarily impairs oxidative phosphorylation efficiency.

Group 2: Disruption of Ion Transport and Plasmalemmal Excitability

Mutations within this category directly alter the baseline electrogenic properties of the synaptic plasma membrane (plasmalemma), shifting the threshold of neuronal excitability and rendering circuits hypersensitive to mechanical shock.

- ***para (bss)*** **(*paralytic / bangsenseless*):** A hypermorphic, gain-of-function (GOF) mutation in the α-subunit of the voltage-gated sodium channel. It causes channel hyperactivation upon stimulation, flooding the neurons with a massive, uncontrolled influx of Na^+ ions.
- ***ATPα*****:** A loss-of-function mutation in the catalytic ***α***-subunit of the Na^+/K^+-ATPase. It directly reduces the pump's capacity to extrude intracellular Na^+ ions and restore the resting membrane potential following depolarization.
- ***jus (sda)*** **(*julius seizure / slamdance*):** A mutation in a specialized two-pass transmembrane scaffold protein. In its absence, the Na^+/K^+-ATPase - ATPase complex loses its structural stability within synaptic lipid rafts and undergoes functional inactivation.

Understudied Group (Baseline Modifier Control)

- ***bas*** **(*bang sensitive*):** The exact molecular identity and biochemical function of this locus remain uncharacterized. It is currently categorized as an understudied baseline modifier of membrane excitability.
